# Thermo-responsive water purification: a thermo-switchable molecular brush for precision engineering of antibacterial ZnO on agro-waste filters

**DOI:** 10.1039/d6ra02207c

**Published:** 2026-05-21

**Authors:** Chuanrui Xia, Xue Wu, Xin Gao, Qiming Li, Lincai Peng, Tian Si, Heng Zhang

**Affiliations:** a Faculty of Chemical Engineering, Kunming University of Science and Technology Kunming 650500 Yunnan China drgaoxin@sina.com 13988202317@163.com zhangheng0625@sina.com; b Dehong Dai and Jingpo Autonomous Prefecture Institute of Sugar Industry Mangshi 678400 Yunnan China

## Abstract

Waterborne pathogens remain a major threat to public health, highlighting the need for sustainable antibacterial filtration materials. Herein, we report a thermo-responsive biomass-based filter constructed by grafting poly(N-acryloyl glycinamide) (PNAGA) molecular brushes onto delignified maize stalk pith (DMSP), followed by *in situ* growth of ZnO nanostructures. The grafted PNAGA layer exhibits upper critical solution temperature (UCST)-type behavior, producing temperature-dependent swelling and interfacial hydration changes that influence ZnO nucleation and growth on the porous scaffold. Compared with ungrafted DMSP, DMSP-*g*-PNAGA promotes more distinct ZnO morphologies under different synthesis temperatures, including needle-like structures at 25 °C and hierarchical flower-like assemblies at 40 °C. HRTEM analysis revealed representative (001)-related lattice fringes in ZDP-T25-t490 and (100)-related lattice fringes in ZDP-T40-t490, while DFT/ESP calculations suggest that temperature-dependent PNAGA conformations alter their preferential interactions with ZnO surfaces. The optimized ZDP-T40-t490 filter achieved high ZnO loading of 659.7 mg g^−1^ and strong initial antibacterial performance, with LRV values of 5.96 ± 0.12 against *Escherichia coli* (*E. coli*) and 5.87 ± 0.15 against *Staphylococcus aureus* (*S. aureus*). The antibacterial activity is attributed to the combined effects of bacterial retention within the labyrinthine pores, Zn^2+^ release, membrane damage, intracellular oxidative stress, and morphology-assisted ZnO-bacteria contact. Repeated-filtration and Zn leaching tests further indicate that long-term stability and initial Zn release remain important limitations. This work provides a proof-of-concept strategy for integrating agro-waste-derived porous scaffolds, UCST-responsive polymer brushes, and antibacterial ZnO nanostructures, while further optimization is required before practical water-treatment application.

## Abstract 2

PNAGA brushes on delignified maize stalk pith (DMSP) regulate ZnO (100) facets into flower-like structures, enabling maze-like entrapment and >99.999% antibacterial inactivation.

## Graphical abstract

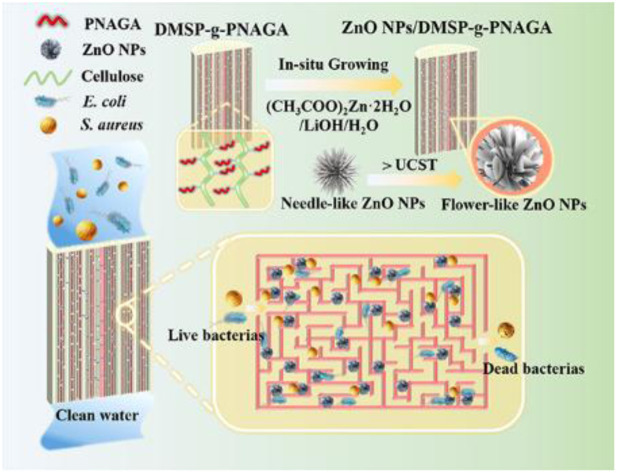

## 1. Introduction

Waterborne bacterial pathogens pose a persistent and severe threat to global public health, disproportionately affecting regions with inadequate sanitation.^1,2^ The World Health Organization estimates that approximately 2.1 billion people consume water contaminated with pathogenic microorganisms, contributing to around 4 billion annual cases of water-related disease and 3.4 million deaths globally.^3–6^ Predominant pathogens like *E. coli* and *S. aureus* cause debilitating illnesses such as diarrheal disease, underscoring the critical need for green, efficient, and safe disinfection technologies.^7,8^ Conventional water purification methods, including chlorination,^9^ ultraviolet irradiation,^10^ and ozonation,^11^ are often hampered by significant drawbacks such as the formation of toxic disinfection byproducts, high energy consumption, and operational costs.^12^ Consequently, the development of sustainable, low-toxicity and potent alternatives remains a paramount challenge.

Inorganic NPs have emerged as a promising platform for next-generation water disinfection. Among them, metal oxide NPs such as ZnO, TiO_2_, and CuO offer compelling advantages, including broad-spectrum antimicrobial activity, low propensity for resistance, and multi-mechanistic action.^13,14^ However, the antibacterial mechanism is critically dependent on intimate contact between the NPs and bacterial cells, which is governed by the NP's crystallographic morphology, size, and dispersion state.^15–17^ For instance, three-dimensional microstructures with high specific surface area can entrap pathogens, while ultrasmall NPs (<10 nm) can readily accumulate within the cell membrane and cytoplasm.^18^ Furthermore, the synthesis of such tailored nanostructures is inherently linked to the reaction environment, where the polarity of the solvent plays a decisive role in directing anisotropic crystal growth by selectively adsorbing to and inhibiting specific crystallographic facets (*e.g.*, polar (001) or non-polar (100) planes), ultimately dictating the final architecture of the ZnO NPs.^19–21^ Therefore, a bifunctional substrate that integrates the controlled growth of optimally structured ZnO with dynamic bacteria capture is crucial for highly efficient disinfection.

Recent studies have also highlighted the importance of green synthesis and biopolymer-assisted stabilization in the development of functional nanomaterials. For example, chitosan-coated selenium nanoparticles and chitosan-coated platinum nanoparticles have been developed as green nanosystems for drug delivery applications, demonstrating that biopolymer coatings can improve nanoparticle stability, dispersibility, and biological performance.^22,23^ In addition, chitosan-coated ZnO nanoparticles have shown antibacterial, antioxidant, and anti-inflammatory potential, further confirming the multifunctional biological activity of ZnO-based nanomaterials.^24^ These studies suggest that polymer/nanomaterial hybrid interfaces are important for regulating nanoparticle stability and bioactivity. However, most reported biopolymer-coated nanomaterials rely on passive stabilization or drug-delivery functions, while fewer studies have explored thermo-responsive polymer brushes as dynamic interfacial regulators for controlling ZnO growth on porous biomass scaffolds. Therefore, constructing an agro-waste-derived scaffold that combines thermoresponsive polymer brushes, *in situ* ZnO growth, and bacterial retention remains attractive for water disinfection applications.

PNAGA is a temperature-responsive polymer exhibiting an UCST behavior, driven by the dual amide groups within its glycinamide side chains, which function as complementary hydrogen bond donors (D) and acceptors (A).^25,26^ Below the UCST, DADA-type hydrogen bond arrays form, leading to non-polar, hydrophobic properties; heating above this transition causes bond dissociation, reflecting polar, hydrophilic characteristics. Meanwhile, the sensitivity of PNAGA to external temperature strongly depends on its molecular weight.^26^ At low molecular weights (5000–40 000), PNAGA displays UCST-type phase separation in water. In contrast, at higher molecular weights (>40 000), it transitions to forming non-thermosensitive, high-strength supramolecular hydrogels with excellent swelling stability.^26^ Hence, to ensure the thermosensitivity of PNAGA, it is rarely homopolymerized into high-molecular-weight hydrogels.

Instead, PNAGA is graft-copolymerized onto polymeric or inorganic substrates, forming thermoresponsive macromolecular brushes with short side chains. For example, Yuan *et al.* incorporated PNAGA into Fe_3_O_4_@GO composite hydrogels, where its reversible hydrogen bonding conferred thermal responsiveness, enabling precise gel–sol transitions and on-demand drug release.^27^ Similarly, Vu *et al.* exploited PNAGA side-chain hydrogen bonding to impart UCST-responsive behavior to alginate hydrogels for temperature-triggered drug delivery.^28^ Building on these insights, we hypothesize harnessing PNAGA's thermo-responsive properties to modulate polymer brush polarity for *in situ* crystal facet engineering of nano-ZnO and antibacterial applications. The limitation of low-molecular-weight PNAGA and small-sized ZnO NPs compromises their ability to concentrate and sterilize highly dispersed bacterial populations in water, leading to significant challenges in accurately evaluating the antibacterial performance of ZnO NPs. Therefore, the selection of target materials for thermosensitive polymer molecular brush grafting is crucial.

In our previous studies, maize stalk pith (MSP)-based columns were developed as anisotropic porous scaffolds for microbial capture and ZnO immobilization.^29–31^ The delignified MSP (DMSP) retains a hierarchical labyrinthine architecture composed of aligned vascular bundles and interconnected pits, which can enhance bacterial retention during filtration.^30^ Herein, we extend this biomass scaffold by grafting UCST-responsive PNAGA molecular brushes onto DMSP, followed by *in situ* growth of ZnO nanostructures. Unlike conventional biomass-supported ZnO filters, where the biomass mainly acts as a passive carrier and bacterial trapper, the grafted PNAGA layer provides a temperature-responsive interfacial environment that affects ZnO loading and morphology. This design integrates three functions within one filtration material: physical bacterial retention by the natural porous scaffold, temperature-dependent interfacial regulation by PNAGA brushes, and antibacterial activity from immobilized ZnO. The resulting ZnO NPs/DMSP-*g*-PNAGA composites were evaluated in terms of thermoresponsive behavior, ZnO growth characteristics, antibacterial performance, preliminary reusability, and Zn leaching.

## 2. Experimental section

### 2.1 Materials

All reagents were analytical grade and used without further purification. Zinc acetate dihydrate (Zn(CH_3_COO)_2_·2H_2_O, ≥99.0%), lithium hydroxide (LiOH, ≥96.0%), acryloyl chloride (≥99.0%), and glycinamide hydrochloride (≥99.0%) were purchased from Aladdin Biochemical Technology Co., Ltd (Shanghai, China). Ultrapure water was used throughout. Melon stalk pith (MSP, bulk density 0.03 g cm^−3^) was manually separated from corn stalks (Yuxi, Yunnan, China), vacuum-dried, and cut into cylinders (diameter 1.5 ± 0.1 cm, length 3.0 ± 0.1 cm). MSP was dewaxed by refluxing in benzene-ethanol (2 : 1 v/v) at 90 °C for 10 h, rinsed with deionized water, lyophilized for 24 h, and delignified with acidic sodium chlorite at 60 °C for 1 h to yield DMSP. Bacterial strains *E. coli* (CCTCC AB 204033) and *S. aureus* (ATCC 25923) were obtained from Kunming University of Science and Technology.

### 2.2 Synthesis of NAGA monomer

Glycinamide hydrochloride (6.3 g) was dissolved in ice water (6 mL). Pre-chilled diethyl ether (18 mL) and K_2_CO_3_ solution (33.6 mL) were added under stirring in an ice bath. Under N_2_, acryloyl chloride in ice-cold diethyl ether was added dropwise at 0 °C. The mixture was warmed to 25 °C and stirred for 4 h. The solution was acidified, extracted with diethyl ether, neutralized with 2 M NaOH, and lyophilized. The crude product was dissolved in ethanol/methanol (4 : 1 v/v), filtered, concentrated by rotary evaporation at 37 °C, and recrystallized at 4 °C for 24 h. Pure NAGA was obtained as a white powder after filtration and freeze-drying.

### 2.3 Preparation of ZnO NPs/DMSP-*g*-PNAGA

DMSP cylinders were sealed in glass tubes. Degassed KPS solution (0.7–2.5 wt% relative to monomer, 0.5 mL) was injected, and the system was preheated at 60 °C for 10 min. NAGA monomer (0.3–3 wt%) was added, and graft polymerization proceeded at 50–70 °C for 18–30 h. The product was washed with deionized water and freeze-dried to yield DMSP-*g*-PNAGA. The grafting yield was derived from eqn (1):^32^1
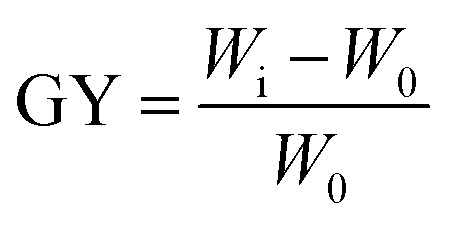
where *W*_0_ and *W*_i_ denote the masses of pristine and grafted DMSP, respectively.

DMSP-*g*-PNAGA was soaked in 2 M zinc acetate at 40 °C for 30 h, then transferred to 0.5 M NaOH and heated sequentially at 25 °C and 40 °C for 8 h each. The composite was rinsed to neutral pH, freeze-dried for 48 h, and thermally treated at 100 °C for 24 h to grow ZnO nanoparticles, yielding ZnO NPs/DMSP-*g*-PNAGA.

### 2.4 Morphological and Componential characterization

Morphology and composition were analyzed by SEM (SU8010, Hitachi), EDS (JAX-8100, JEOL), XRD (X'pert-3, PANalytical), FT-IR (Nicolet), and XPS (Thermo ESCALAB 250Xi). HRTEM was further employed to identify the local lattice fringes of ZnO nanostructures in representative ZDP-T25-t490 and ZDP-T40-t490 samples. Details regarding the calculation of crystallite sizes (D) and crystallinity index (CrI) are provided in the SI. Zinc content was determined by ICP-OES (Optima 8000DV, PerkinElmer). Specific surface area was measured by N_2_ adsorption (BET, ASAP 2020, Micromeritics) after degassing at 85 °C for 12 h. Pore structure was assessed by mercury intrusion porosimetry (Autopore IV 9510). ^1^H NMR and solid-state ^13^C NMR analyses were performed on a Bruker AVANCE NEO-600 spectrometer at 600 MHz, using D_2_O for ^1^H NMR and DMSO-d_6_ for ^13^C-NMR measurements. The zeta potentials of powdered DMSP-*g*-PNAGA and ZnO NPs/DMSP-*g*-PNAGA, diluted to 0.1 wt% aqueous suspensions, were measured at 25 °C using a Mütek SZP-10 Zeta Potential System (BTG, Sweden).

### 2.5 Temperature response performance testing of DMSP-*g*-PNAGA

#### 2.5.1 Determination of swelling degree

The equilibrium swelling ratio (*Q*_*t*_, *Q*_max_) was determined by periodic mass measurements of samples immersed in deionized water, following eqn (2) and (3):^33^2
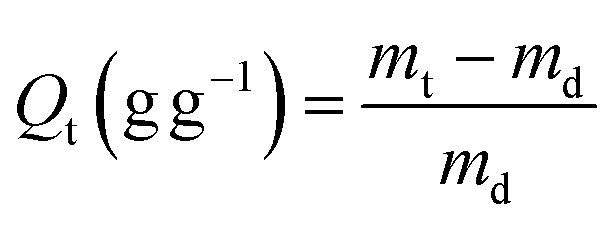
3
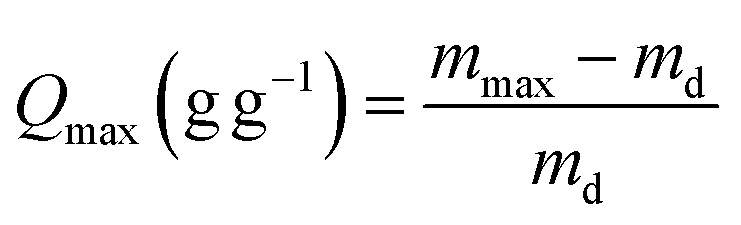
where *m*_*t*_ (g) and *m*_max_ (g) are the weight of samples at swelling time and at swelling equilibrium, respectively; *m*_d_ (g) is the absolutely dry weight of the sample. Each experiment was performed in triplicate to quintuplicate, and the reported values represent the mean.

#### 2.5.2 Kinetics models

Kinetics were analyzed using pseudo-first-order, pseudo-second-order, and intraparticle diffusion models. The corresponding equations are given as (4)–(6):^34^

4 ln(*q*_e_ − *q*_*t*_) = ln *q*_e_ − *k*_1_*t*

5
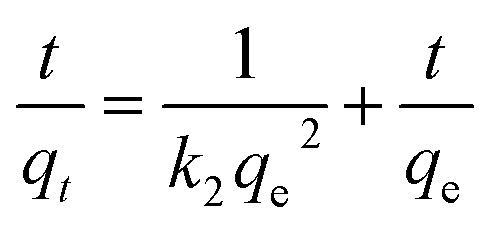

6 *q*_*t*_ = *k*_id_*t*^½^ + *C*_i_

where *k*_1_ (min^−1^), *k*_2_ (g mg^−1^ min^−1^), and *k*_id_ (mg g^−1^ min^−1/2^) are the rate constants for the pseudo-first-order, pseudo-second-order, and intraparticle diffusion models, respectively. The parameter *C*_i_ (mg L^−1^) is the intraparticle diffusion constant, *t* (min) is the adsorption time, while *q*_*t*_ and *q*_e_ (mg g^−1^) represent the adsorption capacities at time *t* and at equilibrium, respectively.

#### 2.5.3 Pure water flux

The thermoresponsive performance of DMSP-*g*-PNAGA was characterized by measuring its pure water flux at various temperatures, calculated using the given equation:^35^7
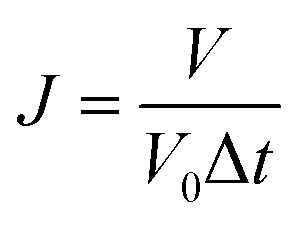
where *J* is the flux of ultrapure water (L m^−3^ h^−1^), *V* is the permeated liquid volume (L), *V*_0_ represents the effective column volume (m^3^); while Δ*t* signifies the time duration of the permeation process (h).

### 2.6 Filter design and assembly

ZnO NPs/DMSP-*g*-PNAGA cylinders were placed in autoclaved glass tubes (50 mm × 16 mm, length × diameter) and sealed with hot-melt adhesive. Tubes were connected *via* ground joints to form adjustable-length 3D filters. The outlet was coupled to a peristaltic pump (NKCP-S10B) for flow control. The system was autoclaved before use.

### 2.7 Microbial inactivation evaluation

*E. coli* and *S. aureus* suspensions (10^7^ CFU mL^−1^) were passed through the filter (500 mL, 10 mL min^−1^). Effluent (100 µL) was plated and incubated at 37 °C for 24 h. Unmodified DMSP served as control. Unless otherwise stated, all antibacterial filtration tests were conducted without intentional external irradiation; photocatalytic disinfection was not evaluated in this study. All tests were performed in a minimum of three replicates, and the antimicrobial efficiency was expressed as the logarithmic removal value (LRV) according to eqn (8):^36^8
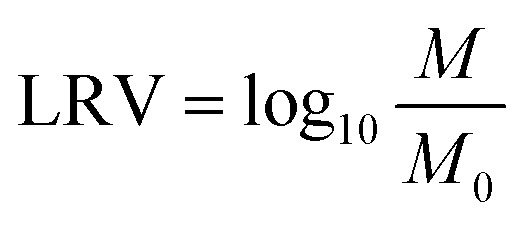
where *M*_0_ and *M* correspond to the bacterial colony counts in the control group and the experimental group, respectively.

### 2.8 Measurement of intracellular oxidative stress by DCFH-DA

Bacterial suspensions were loaded with 10 mM DCFH-DA for 30 min in the dark, washed with PBS, and analyzed by flow cytometry (guava® easyCyte 6–2 L system, Millipore) for DCF fluorescence.^37,38^

### 2.9 Live/dead fluorescent staining of bacteria

Post-treatment bacteria were stained with NucView Green (NV) and propidium iodide (PI) for 15 min, washed, and imaged by fluorescence microscopy (IX83, Olympus).

### 2.10 Antioxidant enzyme assay for bacteria

Bacterial cultures (∼10^8^ CFU mL^−1^) were lysed by sonication. SOD and CAT activities in the supernatant were measured using commercial kits (SOD: Beyotime, Shanghai; CAT: Jiancheng Bioengineering, Nanjing) at 450 nm and 405 nm, respectively.

### 2.11 Measurement of nucleic acid leakage

Bacterial suspensions (*E. coli* and *S. aureus*, 10^8^ CFU mL^−1^) were subjected to filtration through the assembled device for different time intervals (0, 10, 20, 30 min). This was followed by centrifugation (5000 rpm, 5 min) to obtain the supernatant. The supernatant was then diluted 10-fold, and its absorbance was determined with a FlexA-200HT analyzer, enabling the calculation of DNA leakage *via*eqn (9):

9 dsDNA = *n*(OD_260_ − OD_310_) × 50

where OD_260_ and OD_310_ correspond to the optical density measured at 260 nm and 310 nm, respectively, while *n* represents the dilution magnification.

### 2.12 Bacterial genomic DNA testing

DNA was extracted using a commercial kit, separated by 1% agarose gel electrophoresis (60 V, 30 min in 1× TAE), and visualized under UV.

## 3. Results and discussion

### 3.1 Preparation and characterization of ZnO NPs/DMSP-*g*-PNAGA

**Fig. 1:**
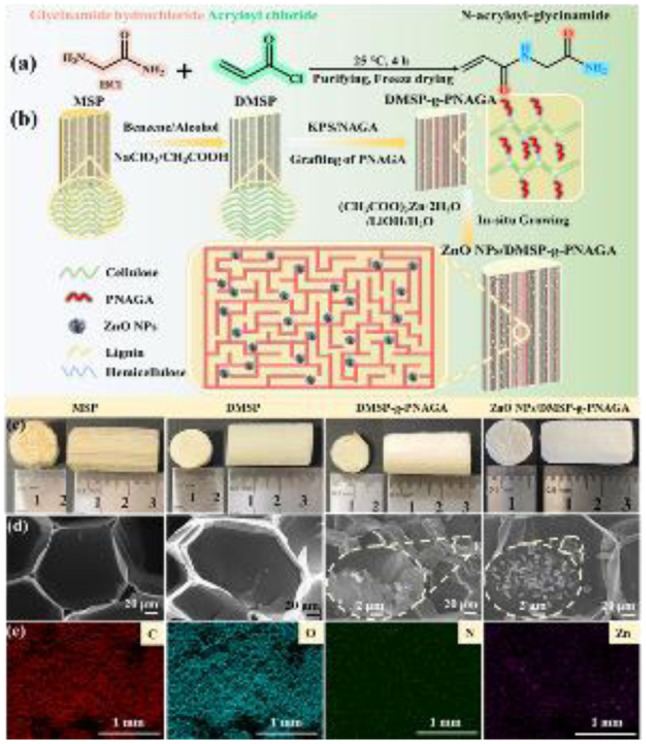
Schematic of (a) NAGA monomer preparation and (b) the top-down approach for preparing ZnO NPs/DMSP-*g*-PNAGA hybrid filtration columns. (c) Digital picture of MSP, DMSP, DMSP-*g*-PNAGA and ZnO NPs/DMSP-*g*-PNAGA. (d) Scanning electron microscopy (SEM) images. (e) energy dispersive spectroscopy (EDS) elemental mapping (C, O, N and Zn) images of ZnO NPs/DMSP-*g*-PNAGA composite filter material (scale bar = 20 µm, 2 µm and 1 µm for (d and e), respectively).

The synthesis of the NAGA monomer and the preparation of the temperature-responsive antibacterial column ZnO NPs/DMSP-*g*-PNAGA are illustrated in Fig. 1a and b. The NAGA monomer was synthesized *via* Schotten–Baumann amidation, in which acryloyl chloride reacts with glycinamide hydrochloride in alkaline aqueous solution through a nucleophilic acyl substitution (addition–elimination) mechanism.^39,40^ Successful synthesis was confirmed by FT-IR, ^1^H NMR, and ^13^C NMR spectra (Fig. S1–S3). MSP features a hierarchical porous structure rich in parenchyma cells, which facilitates efficient transport of water and nutrients and enables *in situ* modification *via* liquid-phase infiltration.^41^ Compared to sclerenchyma-derived wood fibers (specific surface area and density, 2.0–3.3 m^2^ g^−1^ and 92–135 kg m^−3^),^29^ MSP exhibits a higher specific surface area (3.7 m^2^ g^−1^) (Table S1) and lower density (25.7 kg m^−3^),^42^ enhancing reagent accessibility. However, native lignin hinders Zn^2+^ adsorption, risking detachment of ZnO NPs during application.^43,44^ To address this, non-polysaccharide components (lignin, waxes, and lipids) were removed by benzene-ethanol extraction followed by NaClO_2_/CH_3_COOH delignification. Under acidic conditions, NaClO_2_ generates ClO_2_, which selectively cleaves ester bonds between lignin and hemicellulose, as well as ether and C–C bonds within lignin macromolecules, while reacting minimally with carbohydrates.^45^ This process preserves the native columnar porous architecture and increases polysaccharide content from 65.45 to 89.67%,^30^ facilitating subsequent PNAGA grafting and ZnO NPs growth. PNAGA was subsequently grafted onto the DMSP backbone *via* free radical graft copolymerization in aqueous medium, initiated by thermal decomposition of KPS. The resulting sulfate radical anions abstracted hydrogen atoms from cellulose hydroxyl groups, generating cellulose macroradicals that initiated polymerization of NAGA monomers and yielded covalently grafted PNAGA side chains. ZnO nanoparticles were then incorporated into the DMSP-*g*-PNAGA matrix through *in situ* synthesis *via* oriented attachment of ZnO seed crystals. Zn^2+^ ions from zinc acetate were first strongly adsorbed onto the polysaccharide backbone *via* ion–dipole interactions with abundant hydroxyl and diamide groups on the grafted PNAGA chains, creating heterogeneous nucleation sites. The adsorbed Zn^2+^ ions were subsequently transformed into zinc hydroxide intermediates within the lithium hydroxide solution. At elevated temperature, thermal dehydration and decomposition (Zn^2+^ + 2OH^−^ → Zn(OH)_2_ → ZnO + H_2_O) produced anchored wurtzite ZnO seed crystals. Through oriented attachment and stacking, uniformly adherent ZnO NPs grew within the DMSP-*g*-PNAGA matrix, producing the composite filter with a 3D maze network.

The native yellow hue of MSP was removed *via* treatment with acidic NaClO_2_, which generates ClO_2_*in situ* to selectively oxidize and cleave lignin chromophores,^46,47^ yielding a white, morphologically intact DMSP (Fig. 1c). Subsequent *in situ* grafting of PNAGA followed by nucleation and growth of ZnO NPs further enhances surface whiteness. Although lignin removal in DMSP creates pores that introduce minor optical inhomogeneity, PNAGA grafting partially fills and masks these microscopic voids, yielding a smoother and more uniform optical interface. The uniform deposition of ZnO NPs further refines this interface, forming a composite surface layer characterized by enhanced light scattering. Consequently, the average pore size of ZnO NPs/DMSP-*g*-PNAGA is reduced by 6%, 18%, and 10% compared to DMSP-*g*-PNAGA, DMSP, and pristine MSP, respectively (Table S1).

SEM confirms that the honeycomb-like porous architecture is maintained throughout delignification and subsequent modifications (Fig. 1d), with abundant parenchyma cells interconnected by pit channels. The high porosity (98.13%) and intricate labyrinthine network of MSP enhance solution permeation and zinc ion adsorption,^41^ in contrast to natural wood membranes that rely on less efficient vertical channels.^48^ These features render MSP an exceptional scaffold for advanced water disinfection applications. Sub-micrometer particles (<1 µm) are observed on DMSP-*g*-PNAGA (from grafted PNAGA) and ZnO NPs/DMSP-*g*-PNAGA (from in situ-grown ZnO NPs), respectively. Due to enhanced roughness from PNAGA grafting and ZnO NPs growth, the specific surface areas are increased by 17% and 25% compared with DMSP (Table S1), which promotes intimate filter-bacteria contact in water. EDS mapping confirms uniform N and Zn distributions (Fig. 1e), verifying successful PNAGA grafting and homogeneous ZnO NPs growth. This structural design differs from previously reported biomass-supported ZnO filters, where the biomass substrate mainly served as a passive porous support and bacterial trapper. Here, the grafted PNAGA layer additionally provides a thermo-responsive interfacial environment, allowing the scaffold to participate in ZnO growth regulation rather than merely acting as a carrier.

**Fig. 2:**
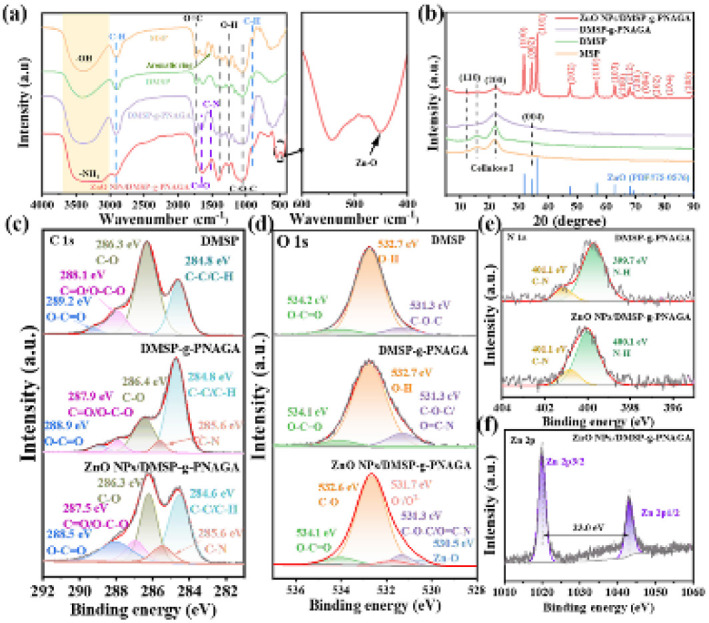
(a) FT-IR spectra, (b) X-ray diffraction (XRD) patterns and high-resolution X-ray photoelectron spectroscopy (XPS) spectra of (c) C 1s, (d) O 1s, (e) N 1s and (f) Zn regions of ZnO NPs/DMSP-*g*-PNAGA along with their controls.

FT-IR spectroscopy was employed to track the chemical evolution throughout the stepwise modification from native MSP to delignified DMSP, grafted DMSP-*g*-PNAGA, and the final hybrid nanocomposite ZnO NPs/DMSP-*g*-PNAGA (Fig. 2a). The spectra of all samples retained broad bands associated with O–H stretching (3413 cm^−1^) and C–H stretching (2930 cm^−1^), characteristic of the lignocellulosic backbone and residual moisture.^49^ In MSP, distinct signals for lignin and hemicellulose, C=O stretching at 1736 cm^−1^ and aromatic C=C vibrations at 1514 cm^−1^ and 1448 cm^−1^, were observed.^41,50^ These peaks were substantially diminished in DMSP, confirming effective delignification and the exposure of reactive hydroxyl groups. Successful grafting of PNAGA onto DMSP was evidenced by the emergence of amide I (C=O, 1650 cm^−1^) and amide II (N–H, 1550 cm^−1^) bands in DMSP-*g*-PNAGA.^51^ FTIR spectra of the ZnO-loaded composites revealed a pronounced decrease in the O–H bending vibration at 1244 cm^−1^, consistent with coordination or electrostatic interactions between Zn^2+^ ions and the cellulosic hydroxyl and PNAGA amide groups. A new band at 468 cm^−1^, assigned to Zn–O stretching, further confirmed the successful *in situ* formation and immobilization of ZnO nanoparticles on the substrate.^52^

XRD analysis elucidated the crystalline evolution of maize stalk pith (MSP) upon delignification and *in situ* modification, as shown in Fig. 2b. Characteristic peaks at 2*θ* = 14.8° (101), 16.5° (101̄), 22.3° (002), and 34.4° (040) in both MSP and delignified MSP (DMSP) confirmed the retention of cellulose I polymorph.^53^ The ZnO NPs/DMSP-*g*-PNAGA composite displayed additional prominent peaks at 31.6° (100), 34.1° (002), 36.5° (101), 47.1° (102), 56.5° (110), 62.8° (103), 67.8° (112), and 70.1° (201), indicative of hexagonal wurtzite ZnO (PDF#75-0576).^18,54^ Complementary FTIR data corroborated the effective immobilization of ZnO nanocrystals on the delignified scaffold without altering the cellulose backbone. Crystallinity index (CrI) and crystallite size varied systematically: MSP exhibited CrI of 34.6% and size of 15.7 nm; delignification increased these to 42.8% and 31.3 nm in DMSP, due to selective removal of amorphous lignin and hemicellulose. PNAGA grafting reduced CrI to 35.3% and size to 29.3 nm, reflecting disrupted cellulose packing. *In situ* ZnO growth reversed this, elevating CrI to 45.6% and size to 33.2 nm, as nanoparticles fostered a more ordered phase. This enhanced crystallinity bolsters mechanical stability, ensuring suitability for water disinfection applications.

The elemental composition and chemical states of DMSP, DMSP-*g*-PNAGA, and ZnO NPs/DMSP-*g*-PNAGA were investigated by XPS. The survey spectra confirmed the introduction of nitrogen after grafting and the presence of zinc in the composite (Fig. S5). High-resolution C 1s spectrum (Fig. 2c) showed an increase in the O/C ratio from 0.45 (DMSP) to 0.62 (DMSP-*g*-PNAGA), consistent with enhanced C–O intensity. The spectrum of DMSP-*g*-PNAGA exhibited a new component at 285.8 eV (C–N) and a stronger O–C=O peak at 288.2 eV, supporting amide formation.^55,56^ In the O 1s spectrum of the composite (Fig. 2d), peaks were assigned to Zn–O–Zn lattice oxygen (530.5 eV), oxygen vacancies/surface –OH (531.7 eV), and C=O/O–C=O species (532.4/533.4 eV).^52,57,58^ The N 1s spectrum of DMSP-*g*-PNAGA (Fig. 2e) showed amide N–C=O (400.1 eV) and N–H (401.4 eV).^55^ After ZnO incorporation, the N–H peak shifted positively by 0.3 eV, suggesting hydrogen bonding or coordination with ZnO. The Zn 2p spectrum (Fig. 2f) displayed symmetric doublets (Zn 2p_3/2_ at 1021.8 eV, Δ = 23.1 eV), characteristic of Zn^2+^ in wurtzite ZnO.^58–60^ These results provide direct evidence for PNAGA grafting and ZnO immobilization through surface interactions, corroborating the structural changes indicated by FTIR and XRD.

### 3.2 Differences in temperature sensitivity

**Fig. 3:**
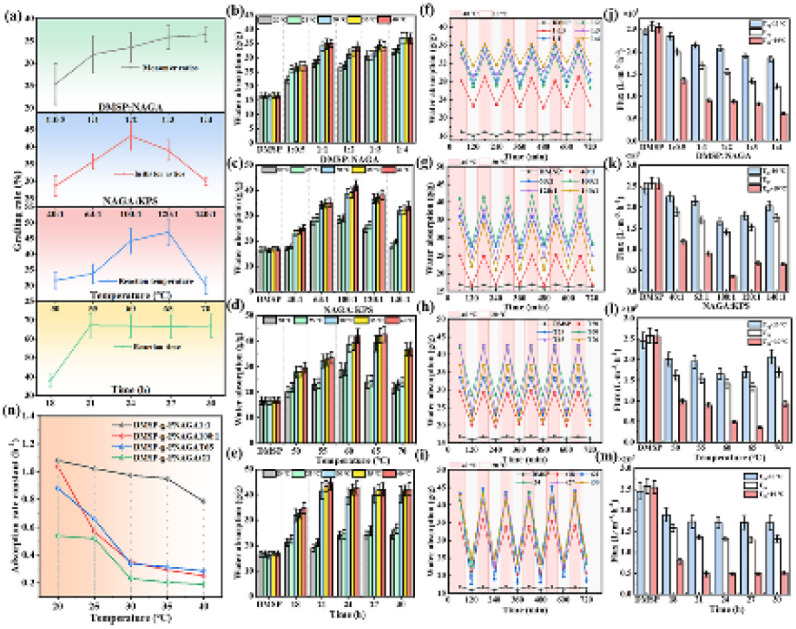
(a) Grafting yields, (b–e) maximum swelling ratios, (f–i) temperature-dependent swelling rates, (j–m) pure water fluxes, (n) pseudo-first-order adsorption equilibrium constants for samples with peak thermosensitivity. All data correspond to DMSP-*g*-PNAGA fabricated with different monomer concentrations, initiator dosages, reaction temperatures, and durations.

The thermoresponsive behavior of DMSP-*g*-PNAGA, governed by synthesis conditions (monomer concentration, initiator dosage, temperature, and time), was systematically evaluated through swelling performance, water flux, and absorption kinetics. Fig. 3a illustrates the dependence of grafting yield on four synthetic variables. Grafting yield steadily increases with rising NAGA monomer concentration, exhibiting the greatest variation when the DMSP : NAGA molar ratio changes from 1 : 0.5 to 1 : 1 (Δ = 6.6%) peaking at 36.3% at a DMSP : NAGA molar ratio of 1 : 4. This trend indicates that the available grafting sites (primarily hydroxyl groups) on the delignified MSP surface approach saturation, while excess monomers promote homopolymerization in solution rather than surface grafting.

The grafting yield displayed a pronounced dependence on initiator concentration, attaining a maximum of 43.2% at a NAGA : KPS molar ratio of 100 : 1, before declining to 30.0% at 140 : 1. This behavior arises from the interplay of radical generation and reaction pathways. At low KPS concentrations, the limited primary radicals preferentially abstract hydrogen from the DMSP surface, creating active sites for grafting. Moderate increases in KPS enhance radical production, thereby improving grafting efficiency. However, excessive initiator promotes rapid homopolymerization of NAGA in solution—a hallmark of persulfate-initiated free-radical systems—consuming monomers and diminishing the fraction available for surface grafting. Thus, the optimal NAGA : KPS ratio of 100 : 1 balances radical initiation with grafting selectivity, maximizing yield for subsequent applications.

Reaction temperature strongly influenced the grafting yield, which reached a maximum of 47.0% at 65 °C. This optimum reflects the accelerated decomposition of KPS at elevated temperatures, generating a higher concentration of sulfate and hydroxyl radicals that initiate grafting. Above 65 °C, however, homopolymerization of NAGA in solution became increasingly competitive, as the greater thermal energy favored chain propagation over surface attachment and thus reduced grafting efficiency.

Grafting yield increased significantly with reaction time, peaking at 67.2% after 21 h before plateauing. Shorter durations (*e.g.*, 18 h: 37.8%) resulted from incomplete initiator decomposition and limited radical sites on DMSP. Extended times offered no gains due to hydroxyl depletion, initiator exhaustion, and steric hindrance. Optimal conditions (NAGA : KPS = 100 : 1, 65 °C, 21 h) maximize density while curbing homopolymerization, enhancing thermoresponsive performance.

The equilibrium swelling ratio of DMSP-*g*-PNAGA was evaluated at 20, 25, 30, 35, and 40 °C across the four synthesis series (Fig. 3b–e). In contrast to the control DMSP, which exhibited relatively stable and low swelling (2–4 g g^−1^), DMSP-*g*-PNAGA displayed pronounced temperature dependence (7–20 g g^−1^), depending on preparation conditions. This thermosensitivity originates from the UCST-type behavior of the grafted PNAGA chains. Below the UCST, strong intra- and interchain hydrogen bonding through DADA arrays of the glycinamide moieties induces collapse of the brushes into a hydrophobic state, thereby limiting water uptake. Above the UCST, dissociation of these hydrogen bonds exposes the polar amide groups, promoting chain extension and enhanced swelling.

The temperature interval producing the largest swelling contrast was selected to identify the most thermosensitive variants (Fig. 3f–i). Maximal swelling differences between 20 °C and 40 °C occurred under the following conditions: DMSP : NAGA = 1 : 1 (Δ = 8 g g^−1^), NAGA : KPS = 100 : 1 (Δ = 14 g g^−1^), 65 °C, and 21 h (Δ values of 19 and 25 g g^−1^, respectively). Notably, these optimal conditions for swelling contrast frequently diverged from those maximizing grafting yield. For instance, grafting yield increased up to a DMSP : NAGA ratio of 1 : 4, yet Δ peaked at 1 : 1. This discrepancy reveals that excessive graft density induces chain crowding and steric hindrance, which restrict PNAGA brush extensibility and thereby dampen the UCST-governed swelling amplitude. Thus, thermosensitive performance reflects a complex balance between graft density and chain mobility.

Pure water flux through DMSP-*g*-PNAGA columns was measured across the UCST transition region. Flux decreased gradually with increasing temperature below the transition, dropped sharply near the UCST, and reached minimum values above it-consistent with collapse of PNAGA brushes into a hydrophobic, less permeable state. Control DMSP exhibited irregular and temperature-insensitive flux. The largest flux contrasts (= 1.1–1.4 × 10^5^ L m^−3^ h^−1^ difference across the transition) were recorded for the same four optimized conditions identified from swelling data, reinforcing that higher grafting density enhances thermoregulated drainability and surface polarity switching.

Water adsorption kinetics were quantified by fitting swelling data to established sorption models (SI Fig. S6–S9 and Tables S2–S9). The pseudo-first-order model afforded the best fit (The value of *R*^2^ is closest to 1), with calculated equilibrium capacities (*q*_e_) in excellent agreement with experimental values (*q*_t_). This indicates that the water absorption process of DMSP-*g*-PNAGA better conforms to the pseudo-first-order kinetic model, and also demonstrates that this process belongs to physical adsorption. The rate constant *k*_1_ decreased systematically with increasing temperature, reaching a minimum of 0.19 h^−1^ under optimal grafting conditions, consistent with retarded water penetration and polymer-chain relaxation in the high-graft-density, thermo-responsive brushes. Intraparticle diffusion analysis (Fig. S6–S9) identified three distinct stages: (i) rapid external surface adsorption (0.1–2.5 h, highest *k*_i1_) onto vascular bundles; (ii) slower intraparticle diffusion (3–4.5 h, decreasing *k*_i2_) through micropores into parenchyma cells; and (iii) equilibrium (5–8 h, lowest *k*_i3_) at saturated sites. The non-zero intercept in the Weber–Morris plot indicates that the swelling process is co-regulated by external surface diffusion and intraparticle diffusion through the intricate, irregular pore network of DMSP-*g*-PNAGA.

Considering both thermoresponsive performance and material cost, the sample prepared at DMSP : NAGA = 1 : 1, NAGA : KPS = 100 : 1, 65 °C, and 21 h was selected for subsequent ZnO growth and antibacterial evaluation. This formulation showed a pronounced temperature-dependent swelling contrast and water-transport response, indicating that the grafted PNAGA brushes can provide a thermally responsive interfacial environment. In the following section, this optimized DMSP-*g*-PNAGA scaffold was used to examine whether PNAGA-associated changes in chain hydration and polar-group accessibility could influence ZnO nucleation, morphology, and preferential crystallographic growth.

### 3.3 Regulation of crystal surface growth in ZnO NPs/DMSP-*g*-PNAGA

To examine whether the UCST-responsive PNAGA brush layer could influence ZnO nucleation and growth, ZnO nanostructures were synthesized on both DMSP and DMSP-*g*-PNAGA under identical temperature and reaction-time conditions. Ungrafted DMSP was used as a control to distinguish the effect of the PNAGA brush layer from that of the biomass scaffold itself.

**Fig. 4:**
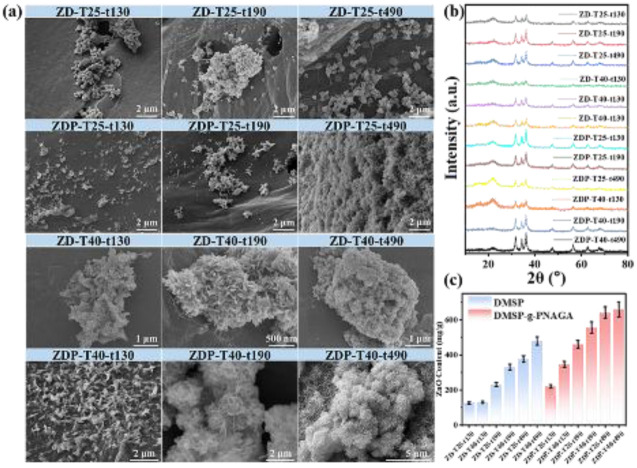
Characterization of ZnO nanoparticles grown *in situ* on DMSP and DMSP-*g*-PNAGA under varied synthesis conditions: (a) SEM images, (b) XRD patterns, and (c) corresponding ZnO content for samples prepared at reaction temperatures of 25 °C and 40 °C and times of 130, 190, and 490 min. (For convenience in describing the materials, ZnO NPs/DMSP will be abbreviated as ZD, and ZnO NPs/DMSP-*g*-PNAGA will be abbreviated as ZDP).

As shown in Fig. 4a, ZnO grown on DMSP-*g*-PNAGA at 25 °C exhibited needle-like nanostructures with diameters of approximately 10 nm, which gradually stacked along their long axes with increasing reaction time. In contrast, ZnO grown on bare DMSP showed irregular aggregates without a clear time-dependent morphological evolution. At 40 °C, ZnO on DMSP-*g*-PNAGA developed into hierarchical flower-like assemblies, whereas ZnO on DMSP remained relatively disordered. These differences indicate that the PNAGA-grafted layer influences ZnO nucleation and growth on the biomass scaffold.

A plausible explanation is that the temperature-dependent hydration state of PNAGA changes the accessibility of amide groups toward Zn-containing intermediates. Below the UCST, stronger intra- and interchain hydrogen bonding may restrict some polar amide groups and favor anisotropic ZnO growth. Above the UCST, enhanced chain hydration and greater exposure of amide groups may strengthen PNAGA-ZnO interfacial interactions, thereby altering the relative growth rates of different ZnO crystal planes and promoting flower-like assembly. This interpretation is supported by the combined SEM, XRD, HRTEM, and DFT/ESP results discussed below, but it should be regarded as a plausible mechanism rather than direct proof of local polarity-controlled crystallization.

XRD analysis confirmed that ZnO grown on both DMSP and DMSP-*g*-PNAGA retained the hexagonal wurtzite structure (Fig. 4b). The relative intensity ratio of the c-axis-related diffraction peak to the (100) peak was used only as a qualitative indicator of crystallographic orientation variation, rather than as direct evidence of exposed surface facets. On ZD, this ratio showed no systematic variation, whereas on ZDP it changed more regularly with reaction time. This trend is consistent with the SEM-observed morphology evolution on the PNAGA-grafted scaffold. However, because XRD mainly reflects bulk crystallographic information and can be affected by preferred orientation, crystallite size, crystallinity, and ZnO loading, the diffraction intensity ratio alone cannot demonstrate surface facet exposure.

The ZnO content of the 12 composites was further quantified by ICP-OES (Fig. 4c). ZnO loading increased with reaction time, and the highest value of 659.7 mg g^−1^ was obtained for ZDP-T40-t490. This result is consistent with the denser flower-like ZnO structures observed by SEM and suggests that the PNAGA-grafted scaffold facilitates ZnO nucleation and growth at 40 °C. Nevertheless, because ZnO loading, morphology, and crystallographic orientation vary simultaneously, these data should be interpreted as supportive evidence for PNAGA-assisted ZnO growth regulation rather than definitive proof of facet engineering.

**Fig. 5:**
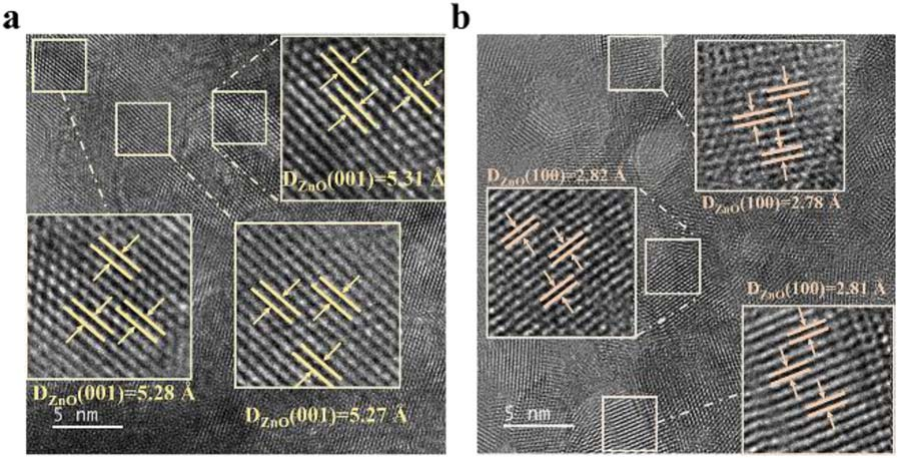
HRTEM images of representative ZDP samples. (a) ZDP-T25-t490 showing lattice fringes with an interplanar spacing of approximately 0.527–0.531 nm, corresponding to the (001) plane of hexagonal wurtzite ZnO. (b) ZDP-T40-t490 showing lattice fringes with an interplanar spacing of approximately 0.278–0.282 nm, corresponding to the (100) plane of ZnO. The HRTEM results provide local crystallographic evidence for the different ZnO growth behaviors at 25 °C and 40 °C.

To provide local crystallographic evidence, HRTEM characterization was performed for two representative samples, ZDP-T25-t490 and ZDP-T40-t490 (Fig. 5). For ZDP-T25-t490, clear lattice fringes with an interplanar spacing of approximately 5.27–5.31 Å were observed, which can be indexed to the (001) plane of hexagonal wurtzite ZnO. This c-axis-related lattice feature is consistent with the needle-like ZnO morphology formed at 25 °C.

In comparison, ZDP-T40-t490 exhibited hierarchical flower-like ZnO assemblies. Multiple local regions showed lattice fringes with interplanar spacings of approximately 2.78–2.82 Å, corresponding to the (100) plane of ZnO. The slight variation in fringe orientation among different regions is reasonable considering the assembled and polycrystalline nature of the flower-like ZnO nanostructures. These HRTEM observations complement the SEM and XRD results and support the conclusion that the PNAGA-grafted scaffold influences ZnO morphology and preferential crystallographic growth. However, the HRTEM results should not be interpreted as quantitative evidence for the fraction of exposed surface facets.

**Fig. 6:**
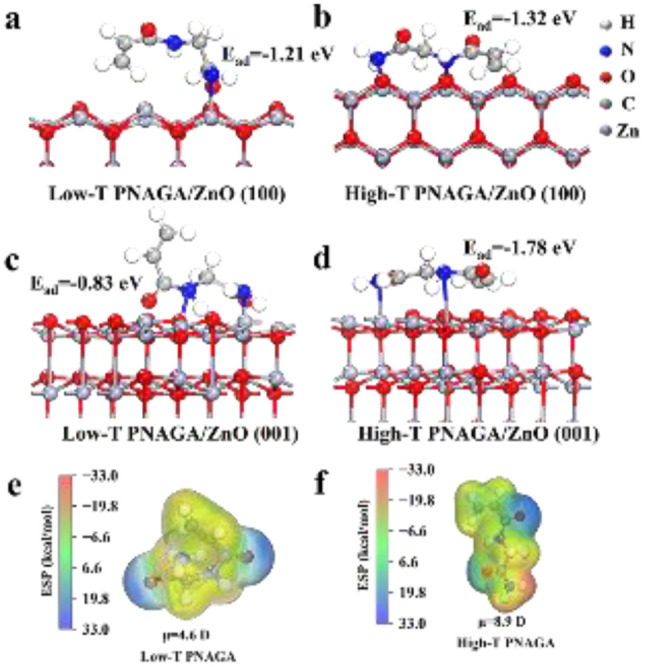
ESP analysis from DFT calculation of temperature-dependent PNAGA-ZnO interactions. Optimized adsorption configurations of (a) low-T PNAGA on ZnO-(100), (b) high-T PNAGA on ZnO-(100), (c) low-T PNAGA on ZnO-(001), and (d) high-T PNAGA on ZnO-(001). ESP maps of (e) low-T and (f) high-T PNAGA segments, showing dipole moments of 4.6 and 8.9 D, respectively. The ESP maps were plotted using the same electrostatic potential scale.

To further provide molecular-level support for the temperature-regulated ZnO growth mechanism, DFT calculations were performed to evaluate the adsorption behavior of PNAGA segments on ZnO-(100) and ZnO-(001) surfaces. The adsorption energy was calculated as *E*_ads_ = *E*_PNAGA/ZnO_ −*E*_PNAGA_ − *E*_ZnO_, where a more negative value indicates stronger interfacial interaction. As shown in Fig. 6a–d, the adsorption energies of low-T PNAGA/(100), high-T PNAGA/(100), low-T PNAGA/(001), and high-T PNAGA/(001) were −1.21, −1.32, −0.83, and −1.78 eV, respectively. In the low-temperature state, PNAGA exhibited stronger adsorption on ZnO-(100) than on ZnO-(001), with *E*_ads_(001) − *E*_ads_(100) = +0.38 eV. While, in the high-temperature state, PNAGA showed the strongest adsorption on ZnO-(001), and the adsorption selectivity changed to −0.46 eV. This result suggests that the temperature-induced conformational/polarity change of PNAGA's ureido groups alters its preferential interaction with different ZnO surfaces.

Analyses of ESP and dipole moment further supported the enhanced polarity of high-T PNAGA. As shown in Fig. 6e and f, the dipole moment increased from 4.6 D for low-T PNAGA to 8.9 D for high-T PNAGA, indicating a significantly enhanced molecular polarity after thermal disruption of intra/intermolecular hydrogen bonding. The high-T PNAGA segment exhibited more exposed electropositive and electronegative regions, mainly associated with the amide N–H and carbonyl's oxygen atoms, respectively. Moreover, the ESP maps of the PNAGA/ZnO complexes were further provided in Fig. S10. Among the four adsorption systems, high-T PNAGA/(001) displayed the most pronounced interfacial electrostatic complementarity, where the electronegative carbonyl's oxygen region was close to surface Zn sites and the electropositive N–H region was oriented toward surface O atoms. This interfacial ESP distribution is consistent with the strongest adsorption energy of high-T PNAGA/(001).

These theoretical results indicate that high-temperature PNAGA can preferentially adsorb on and stabilize the polar ZnO-(001) surface. Such preferential adsorption may suppress growth along the [001] direction and promote lateral growth or assembly,^61^ which is consistent with the experimentally observed formation of hierarchical flower-like ZnO at 40 °C. Therefore, the UCST behavior of PNAGA is considered to provide a temperature-dependent interfacial polarity and adsorption environment that modulates ZnO growth behavior.

### 3.4 Antibacterial properties and mechanism of ZnO NPs/DMSP-*g*-PNAGA

**Fig. 7:**
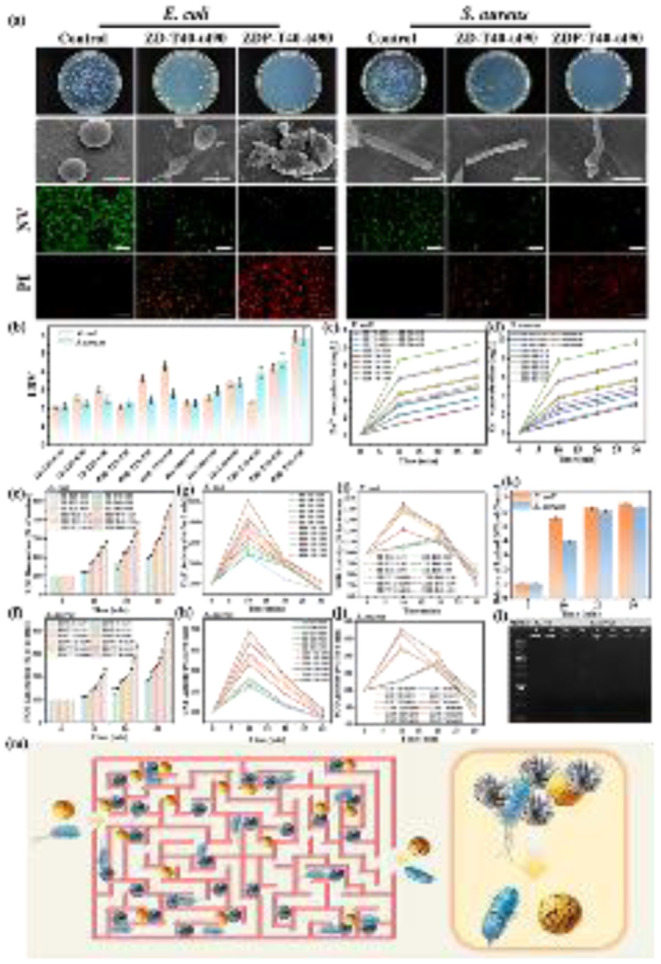
Antibacterial activity and mechanism of synthesized materials. (a) Agar plate photographs of bacterial colonies of *S. aureus* and *E. coli* after exposure to different materials, with corresponding bio-SEM (Scale bar, 500 nm) and fluorescence images of different materials after incubation under the same conditions, showing live bacteria (green) and dead bacteria (red) (Scale bar: 40 µm). (b) The antibacterial rates of different materials against *E. coli* and *S. aureus.* (c and d) Changes in Zn^2+^ concentration within the treatment solution as a function of sterilization time. The relatively (e and f) intracellular DCF fluorescence intensity, (g and h) CAT activities, (i and j) SOD activities in *E. coli* and *S. aureus* with different materials. (k) The leakage level of nucleic acid for *E. coli* and *S. aureus* after filtration treatment with ZDP-T40-t490. (l) DNA agarose gel electrophoresis of *E. coli* and *S. aureus* exposed to ZDP-T40-t490. (m) Schematic representation of the antibacterial mechanism of ZnO-nanomaterials.

The antibacterial and oxidative-stress-related activities of ZnO-based nanomaterials have also been reported in other polymer-coated ZnO systems. For instance, chitosan-coated ZnO nanoparticles were shown to possess antibacterial and antioxidant potential,^24^ which is consistent with the contribution of Zn-related activity and oxidative stress pathways observed in the present ZDP-T40-t490 system. To investigate the antibacterial effects of ZnO NPs grown on different crystal planes, we conducted antibacterial performance and mechanism studies. Fig. 7a shows representative agar plate photographs of surviving bacterial colonies post-filtration, alongside bio-SEM images of bacterial morphology and live/dead fluorescence staining (NV for intact cells, PI red for compromised membranes). Among all DMSP-supported samples, the multi-shaped ZnO synthesized at 40 °C for 490 min (denoted ZD-T40-t490) exhibited the highest antibacterial activity, with LRV values of 3.38 ± 0.11 against *E. coli* and 3.42 ± 0.09 against *S. aureus* (Fig. 7b), corresponding to >99.9% inactivation. Among all DMSP-*g*-PNAGA-supported samples, the flower-like ZnO synthesized at 40 °C for 490 min (denoted ZDP-T40-t490) showed the highest activity, with LRV values of 5.96 ± 0.12 against *E. coli* and 5.87 ± 0.15 against *S. aureus* (Fig. 7b), corresponding to >99.999% inactivation. Notably, ZDP-T40-t490 exhibited substantially higher absolute antibacterial activity than ZD-T40-t490. However, because these two samples differ in both ZnO loading and morphology, the enhanced activity should not be attributed solely to morphology or facet regulation. The ZnO loading of ZDP-T40-t490 was 659.7 mg g^−1^, which was higher than that of ZD-T40-t490, 479.3 mg g^−1^. Therefore, the superior antibacterial performance of ZDP-T40-t490 is more reasonably interpreted as the combined result of higher ZnO loading and flower-like ZnO morphology. The higher ZnO content provides more antibacterial components, while the flower-like morphology may improve bacteria-ZnO interfacial contact and facilitate local membrane damage. Nevertheless, because ZnO loading and morphology vary simultaneously in the present comparison, their individual contributions cannot be completely decoupled based solely on these two samples.

To further clarify the contribution of ZnO loading to antibacterial performance, all ZnO-loaded samples were subjected to a semi-quantitative comparative analysis. Scatter plots of LRV *versus* ZnO loading were constructed for both *E. coli* and *S. aureus* (Fig. S11a and b). For *E. coli*, both ZD and ZDP series exhibited relatively strong positive correlations, with *R*^2^ values of approximately 0.9 (Table S10), confirming that ZnO loading is an important factor governing antibacterial activity. For *S. aureus*, the ZD series showed a moderate correlation (*R*^2^ = 0.7522), whereas the ZDP series displayed a much weaker linear dependence (*R*^2^ = 0.2973), suggesting that total ZnO loading alone cannot fully describe the antibacterial response of the ZDP samples.

In addition, an apparent loading-normalized antibacterial index was calculated by dividing the LRV by the ZnO loading (Fig. S11c). The ZDP series showed lower LRV/ZnO loading values than the ZD series, indicating that the superior absolute LRV of ZDP-T40-t490 should not be interpreted as higher antibacterial activity per unit total ZnO loading. Instead, the enhanced disinfection performance of ZDP-T40-t490 is more reasonably attributed to the combined effects of higher ZnO loading, flower-like ZnO morphology, improved bacteria-ZnO interfacial contact, and the physical retention effect of the porous scaffold. It should be noted that this normalization is semi-quantitative because LRV is a logarithmic parameter and total ZnO loading does not directly represent the exposed active ZnO surface or the bacteria-accessible ZnO fraction.

Fluorescence images revealed predominant green signals in controls, indicating viable cells with intact membranes, whereas ZDP-T40-t490-treated samples showed extensive red fluorescence, signifying membrane permeabilization. Bio-SEM further depicted control bacteria with smooth and intact surfaces, while treated cells displayed deformation, pitting, and lysis, particularly after exposure to ZDP-T40-t490 and ZD-T40-t490. These observations suggest that the maze-like structure of the scaffold may extend bacterial retention time and increase the probability of bacteria contacting immobilized ZnO nanoparticles. In addition, the flower-like ZnO morphology in ZDP-T40-t490 may provide more accessible contact sites than irregularly aggregated ZnO, thereby facilitating local membrane damage. However, this morphological contribution should be considered together with the higher ZnO loading of ZDP-T40-t490 rather than as an isolated factor. To obtain mechanistic clues for the antibacterial process, several experimentally accessible indicators were examined, including Zn^2+^ release, membrane integrity, intracellular DCF fluorescence, antioxidant enzyme activities, nucleic acid leakage, and genomic DNA integrity. Fig. 7c and d illustrate Zn^2+^ concentrations in the effluent as a function of filtration time (0–30 min). ZDP-T40-t490 and ZD-T40-t490 released the highest levels (up to 1.2–1.5 mg L^−1^ for *E. coli* and 1.0–1.3 mg L^−1^ for *S. aureus* at 30 min), correlating with their superior LRVs. Flower-like ZnO exhibited sustained release kinetics compared to needles, possibly due to higher surface area facilitating dissolution (ZnO + H_2_O → Zn^2+^ + 2OH^−^).^62,63^ Elevated Zn^2+^ disrupts membrane integrity, inhibits enzymatic activity, and binds thiol groups in proteins, contributing to cytotoxicity.^64^

To further evaluate the relationship between Zn^2+^ release and antibacterial activity, the Zn^2+^ concentration at 30 min was plotted against the corresponding LRV values for all ZnO-loaded samples (Fig. S11). Linear fitting was performed separately for the ZD and ZDP series. For *E. coli*, strong positive correlations were observed for both ZD and ZDP, with *R*^2^ values of 0.9123 and 0.9653 (Table S11), respectively, indicating that Zn^2+^ release was strongly associated with *E. coli* inactivation. For *S. aureus*, the correlations were weaker, with *R*^2^ values of 0.5380 for ZD and 0.7151 for ZDP (Table S11). This result suggests that Zn^2+^ release contributes to *S. aureus* inactivation but does not fully determine the antibacterial performance. Therefore, Zn^2+^ release should be considered as one important contributor to bacterial inactivation, together with membrane disruption, intracellular oxidative stress, antioxidant enzyme imbalance, nucleic acid leakage, and bacteria-material contact within the porous scaffold.

Intracellular oxidative stress was evaluated using the DCFH-DA assay (Fig. 7e and f). The treated bacteria showed increased intracellular DCF fluorescence compared with the control groups, indicating that bacterial cells experienced enhanced oxidative stress after filtration through the ZnO-loaded materials. It should be emphasized that DCFH-DA is a general oxidative stress probe and cannot identify specific ROS species. This interpretation is consistent with our previous EPR results, in which exogenous ROS in the aqueous effluent were not detected or were below the detection limit.^29^ Thus, the increased DCF fluorescence observed here should be interpreted as intracellular oxidative stress associated with membrane damage, Zn^2+^ exposure, and cellular stress responses, rather than as evidence of extracellular ROS generation or a photocatalytic ROS-dominated mechanism.

The changes in antioxidant enzyme activities further support the occurrence of oxidative-stress-related cellular responses (Fig. 7g–j). After 10 min of filtration, SOD and CAT activities increased by approximately 1.5–2.0 times compared with the control groups, suggesting an early compensatory response to intracellular oxidative stress. After 30 min, the activities decreased to approximately 40–60% of the baseline values. This decrease may be associated with severe membrane damage, intracellular leakage, and enzyme dysfunction under prolonged exposure to the ZnO-loaded materials and released Zn^2+^. These results support the involvement of intracellular oxidative stress but do not identify specific ROS species.

Zeta potential shifted from −7.27 mV in DMSP to +5.73 mV post-ZnO loading (isoelectric point pH 5.62; Fig. S10), enhancing electrostatic attraction to negatively charged bacterial surfaces and contact killing.^65^ Nucleic acid leakage, quantified by OD_260_ absorbance, escalated progressively with filtration time for ZDP-T40-t490 (reaching 0.45–0.55 AU at 30 min; Fig. 7k), evidencing cytoplasmic release from membrane disruption. Agarose gel electrophoresis (Fig. 7l) displayed smeared and weakened genomic DNA bands after treatment compared with the sharp bands of the controls, suggesting damage to genomic DNA integrity. Because specific ROS species were not directly identified in this study, the DNA damage is more cautiously attributed to the combined effects of membrane disruption, Zn^2+^-associated stress, and intracellular oxidative stress rather than to a confirmed ROS-specific degradation pathway.^66^ Collectively, these results indicate that ZDP-T40-t490 achieves superior absolute disinfection performance through the synergistic integration of scaffold-assisted bacterial retention, high ZnO loading, flower-like ZnO morphology, Zn^2+^ release, intracellular oxidative stress, and membrane/nucleic-acid damage. However, because ZnO loading and morphology change simultaneously among the compared samples, the present data do not allow their individual contributions to be completely separated.

As illustrated in Fig. 7m, the antibacterial action of ZnO NPs/DMSP-*g*-PNAGA can be described as a multi-factor process involving physical retention by the maze-like scaffold, enhanced bacteria–material contact, electrostatic adhesion, membrane disruption, Zn^2+^-associated stress, intracellular ROS accumulation, SOD/CAT imbalance, and DNA/protein damage. Therefore, the enhanced performance of ZDP-T40-t490 should be interpreted as a combined effect of ZnO loading and morphology-assisted interfacial contact rather than as a result of morphology or facet control alone.

### 3.5 Reusability and Zn leaching evaluation

**Fig. 8:**
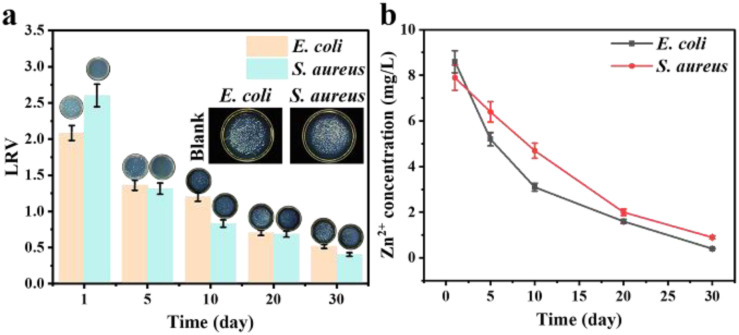
Reusability and Zn-leaching behavior of ZDP-T40-t490 during repeated filtration. (a) Antibacterial LRV values and representative agar plate photographs for *E. coli* and *S. aureus* after repeated filtration using the same ZDP-T40-t490 filter over 30 days. (b) Zn concentration in the post-filtration effluent collected after filtering *E. coli* or *S. aureus* suspensions at different reuse times.

To further evaluate the practical stability of ZDP-T40-t490, the repeated filtration tests were conducted over 30 days using *E. coli* and *S. aureus* suspensions. The antibacterial activity gradually decreased with repeated utilization (Fig. 8a). For *E. coli*, the LRV decreased from 2.08 on the first day to 0.51 on the day 30th. For *S. aureus*, the LRV decreased from 2.60 to 0.40 over the same period. These results indicate that ZDP-T40-t490 retained partial antibacterial activity during repeated filtration, but its long-term antibacterial efficiency declined substantially.

Zn leaching was also monitored in the effluent during repeated filtration. The Zn concentration decreased from 8.6 to 0.4 mg L^−1^ for the filtered *E. coli* suspension and from 7.9 to 0.9 mg L^−1^ for the treated *S. aureus* suspension over 30 days (Fig. 8b). The high Zn concentration at the early stage suggests that loosely bound or surface-accessible Zn species were released during initial filtration. The subsequent decrease in Zn concentration indicates improved leaching stability after repeated washing/filtration, but also coincides with the decrease in antibacterial activity. This parallel trend suggests that dissolved Zn species may contribute to bacterial inactivation, together with contact-mediated antibacterial effects from immobilized ZnO.

These results represent a preliminary reusability and leaching assessment rather than full validation for practical drinking-water application. The initial Zn release was relatively high, indicating that further optimization, such as improved ZnO immobilization, pre-rinsing, or post-treatment, is required to reduce metal leaching while maintaining antibacterial activity.

## 4. Conclusion

In summary, a thermo-responsive antibacterial filter was developed by grafting UCST-active PNAGA molecular brushes onto delignified maize stalk pith, followed by *in situ* growth of ZnO nanostructures. The DMSP scaffold provides a natural labyrinthine porous network for bacterial retention, while the PNAGA layer introduces temperature-dependent swelling and interfacial hydration changes that influence ZnO nucleation and growth. Compared with ungrafted DMSP, DMSP-*g*-PNAGA produced more distinct ZnO morphologies under different synthesis temperatures. SEM, XRD, HRTEM, and DFT/ESP results suggest that PNAGA-assisted synthesis modulates ZnO morphology and preferential crystallographic growth, although exposed facet fractions and local polarity changes were not directly quantified. The optimized ZDP-T40-t490 sample achieved a ZnO loading of 659.7 mg g^−1^ and strong initial antibacterial activity, with LRV values of 5.96 ± 0.12 against *E. coli* and 5.87 ± 0.15 against *S. aureus*. The activity arises from the combined effects of bacterial retention, ZnO loading, flower-like morphology, Zn^2+^ release, membrane damage, and intracellular oxidative stress. Repeated-filtration and Zn leaching tests indicate that long-term stability and initial Zn release require further optimization before practical application.

## Author contributions

Conceptualization, X. Gao; resources, Q. M. Li; supervision, C. R. Xia; writing-original draft, C. R. Xia; writing-review & editing, X. Gao, H. Zhang, L. C. Peng and T. Si. All authors reviewed and approved the final manuscript.

## Conflicts of interest

There are no conflicts to declare.

## Supplementary Material

RA-016-D6RA02207C-s001

## Data Availability

All data generated or analyzed during this study are included in this published article and its supplementary information (SI). Supplementary information: additional experimental and computational methods, NAGA monomer characterization, BET and XPS characterization, swelling kinetic fitting curves and parameters, ESP maps and DFT-related analysis of PNAGA/ZnO interactions, semi-quantitative analyses of ZnO loading-LRV and Zn^2+^ release-LRV relationships, zeta potential data, and additional fitting parameters and *r* values supporting the discussion of ZnO growth and antibacterial performance. See DOI: https://doi.org/10.1039/d6ra02207c.
