# Supplementary material for: Thermo-responsive water purification: a thermo-switchable molecular brush for precision engineering of antibacterial ZnO on agro-waste filters
Source: RSC Adv. 2026 May 21;16(30):27504–17. doi: 10.1039/d6ra02207c (PMC13195426; doi:10.1039/d6ra02207c)
Supplement: RA-016-D6RA02207C-s001 [file RA-016-D6RA02207C-s001.pdf]

Supplementary Information (SI) for RSC Advances.
This journal is © The Royal Society of Chemistry 2026

Supporting Information

Thermo-Responsive Water Purification: A Thermo-Switchable Molecular Brush for Precision Engineering of Antibacterial ZnO on Agro-Waste Filters

Chuanrui Xia,[a] Xue Wu,[a] Xin Gao,*[a] Qiming Li,*[b] Lincai Peng,*[a] Tian Si,[a] Heng Zhang*[a]

[a] Faculty of Chemical Engineering, Kunming University of Science and Technology, Kunming 650500, Yunnan, China

[b] Dehong Dai and Jingpo Autonomous Prefecture Institute of Sugar Industry, Mangshi 678400, Yunnan, China

E-mail: drgaoxin@sina.com; 13988202317@163.com; zhangheng0625@sina.com

Text S1 Effect of PNAGA on the growth of ZnO NPs Crystal Facets

To further corroborate the facet-selective growth, XRD analysis was performed. The relative exposure of crystal facets in ZnO NPs is quantitatively reflected by the intensity ratio (r) = I_{100}/I_{001}.[1] Values of r > 1 indicate predominant growth perpendicular to the [001] direction (enhanced (100) facet exposure), characteristic of flower-like needle-like morphologies, whereas r < 1 signifies preferential elongation along [001] (greater (001) facet exposure), consistent with needle-like habits.

Text S2 Supplementary Experimental Methods

2.1. Calculation of crystal size and crystallinity index

Crystalline phases within the samples were identified with an X'Pert-3 powder diffractometer (PANalytical, Netherlands). Crystallite sizes (D) perpendicular to the (002) lattice plane of cellulose I and the (101) plane of ZnO NPs were calculated using Scherrer's equation (S1):[2]

$$D = \frac{k\lambda}{\beta_{1/2}\cos\theta} \quad \text{(S1)}$$

where k=0.943, λ=0.154056 nm, and $\beta_{1/2}$ represents the full width at half-maximum (4.21×10^{-2} rad for cellulose (002) and 7.80×10^{-3} rad for ZnO (101)).

The crystallinity index (*CrI*) of cellulose I was determined from peak deconvolution according to Eq. (S2):[3]

$$CrI(\%) = \frac{I_{002} - I_{am}}{I_{002}} \times 100\% \quad \text{(S2)}$$

where I_{002} is the intensity of the (002) peak at 2θ=22.3° and I_{am} is the minimum intensity near 2θ=18°. XRD patterns were analyzed using Jade 6.0 software without background subtraction.

2.2. Computational details

Density functional theory (DFT) calculations were performed using the DMol^3 module in Materials Studio to investigate the temperature-dependent interactions

between PNAGA segments and ZnO surfaces. The exchange–correlation interaction was described using the Perdew–Burke–Ernzerhof functional within the generalized gradient approximation (GGA-PBE). Ultrasoft pseudopotentials were used to describe the core–electron interactions. The plane-wave cutoff energy was set to 400 eV, and geometry optimization was performed until the residual force on each atom was less than 0.03 eV $Å^{-1}$.[4]

The bulk hexagonal wurtzite ZnO structure was first optimized, and the optimized lattice parameters were then used to construct ZnO surface slab models. The non-polar ZnO-(100) and polar ZnO-(001) surfaces were modeled using periodic slab models. A vacuum layer of 15 Å was introduced along the surface-normal direction to avoid artificial interactions between neighboring periodic slabs.[5,6] A Monkhorst-Pack k-point mesh of 2 × 2 × 1 was used for the ZnO surface calculations.[6] During geometry optimization, the bottom layer of the ZnO slab was fixed to maintain the bulk-like structure, whereas the upper surface atoms and PNAGA segments were fully relaxed. For the polar ZnO-(001) surface, dipole correction was applied along the surface-normal direction.

To represent the thermoresponsive states of PNAGA, two PNAGA segment models were constructed. The low-temperature PNAGA model was built in a hydrogen-bonded/collapsed conformation, in which part of the amide groups were involved in intra/intermolecular hydrogen bonding. The high-temperature PNAGA model was built in a hydrogen-bond-dissociated/extended conformation, exposing more amide carbonyl and N–H groups toward the ZnO surface. Before adsorption calculations, the isolated PNAGA segments and ZnO slab models were fully optimized using the same calculation parameters. The adsorption energy was calculated according to:

$$E_{ads} = E_{PNAGA/ZnO} - E_{PNAGA} - E_{ZnO} \quad (S2)$$

where $E_{PNAGA/ZnO}$, E_{PNAGA}, and E_{ZnO} are the total energies of the PNAGA/ZnO adsorption complex, isolated PNAGA segment, and clean ZnO slab, respectively. A more negative E_{ads} value indicates a stronger PNAGA-ZnO interaction.

Electrostatic potential (ESP) maps were generated from the optimized electronic structures to visualize the polarity distribution of isolated PNAGA segments and the interfacial electrostatic distribution of PNAGA/ZnO complexes. The dipole moments of the isolated low-temperature and high-temperature PNAGA segments were calculated from the optimized electronic structures. All ESP maps were plotted using the same electrostatic potential scale for direct comparison.

S3 Characterization of NAGA monomer

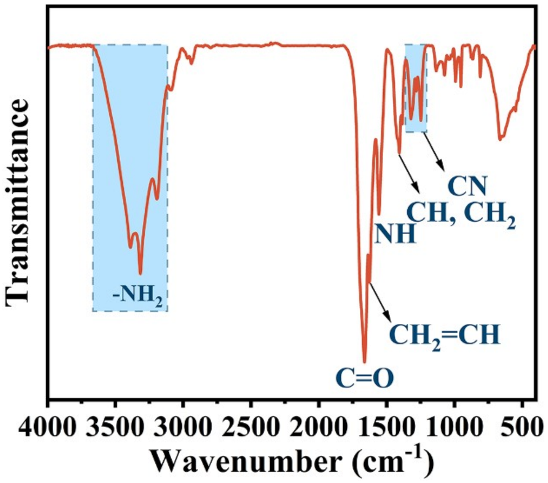

Figure S1 FT-IR spectra of NAGA. FT-IR spectroscopy confirmed successful synthesis of N-acryloyl glycinamide, showing characteristic bands at 3390, 3317, and 3194 cm^{-1} (N-H stretching with hydrogen bonding), 1662 cm^{-1} (amide I, C=O stretching), 1625 cm^{-1} (C=C stretching of vinyl group), 1558 cm^{-1} (amide II), 1408 cm^{-1} (CH_2 bending), and 1258 cm^{-1} (C-N stretching).

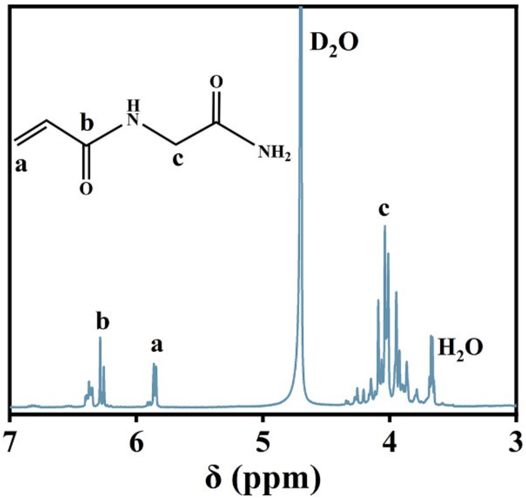

Figure S2 Figure S1 ^{1}H-NMR spectra of NAGA. ^{1}H NMR spectroscopy confirmed the successful synthesis of N-acryloyl glycinamide, displaying characteristic signals at δ 3.9 ppm (2H, NH-CH_2-CONH), 5.8 ppm (2H, CH=CH_2), 6.2-6.3 ppm (1H, CH=CH_2), along with a residual solvent peak at 4.7 ppm. The integrated peak areas were consistent with the expected molecular structure.

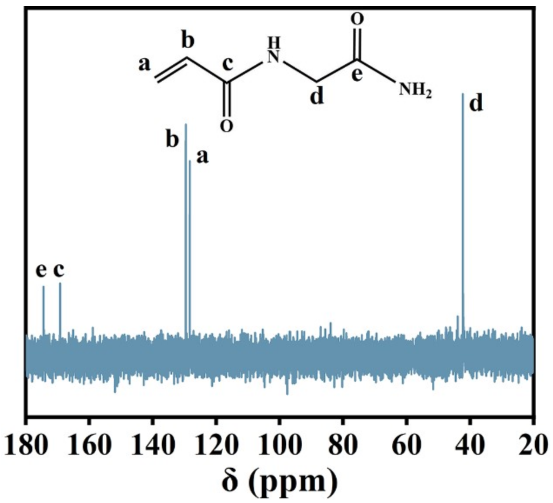

Figure S3 ^{13}C-NMR spectra of NAGA. ^{13}C NMR spectroscopy confirmed the structure of N-acryloyl glycinamide, with characteristic signals at δ 42.3 ppm (-NH-CH_2-CO-), 128.3 and 129.6 ppm (CH=CH_2), 169.2 ppm (-CO-NH- acryloyl carbonyl), and 174.4 ppm (-CONH- glycinamide carbonyl), consistent with the expected molecular structure.

S4 The microstructure of the composite column

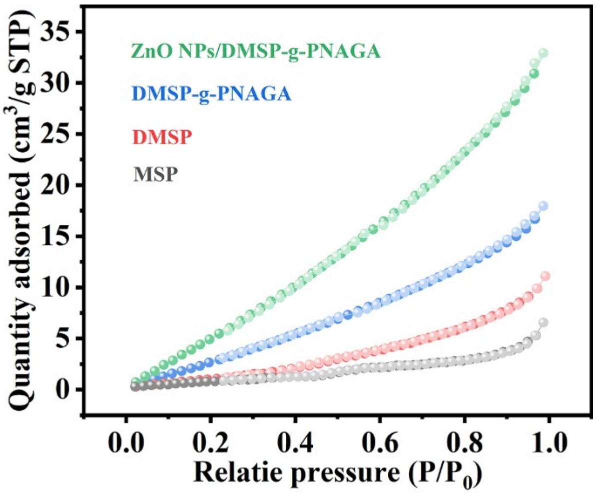

Figure S4 Nitrogen adsorption isotherms of MSP, DMSP, DMSP-g-PNAGA and ZnO NPs/DMSP-g-PNAGA. N_2 adsorption-desorption isotherms revealed that both samples exhibited Type II isotherm behavior characteristic of macroporous structures, featuring reversible adsorption-desorption cycles at high relative pressures without a distinct plateau.

S5 XPS survey spectra of the composite column

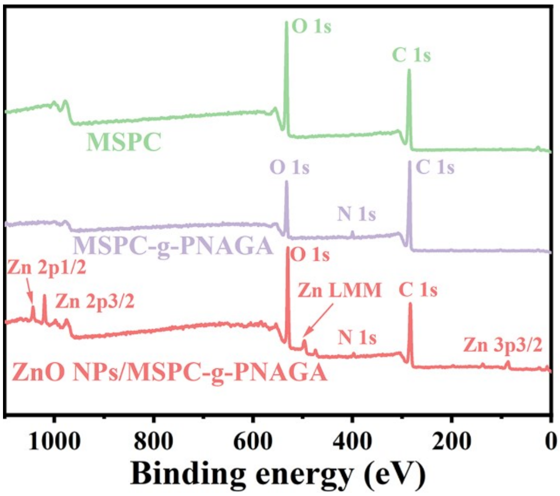

Figure S5 XPS survey spectra of DMSP, DMSP-g-PNAGA and ZnO NPs/DMSP-g-PNAGA.

S6 Pseudo-first-order, pseudo-second-order, and intraparticle diffusion models

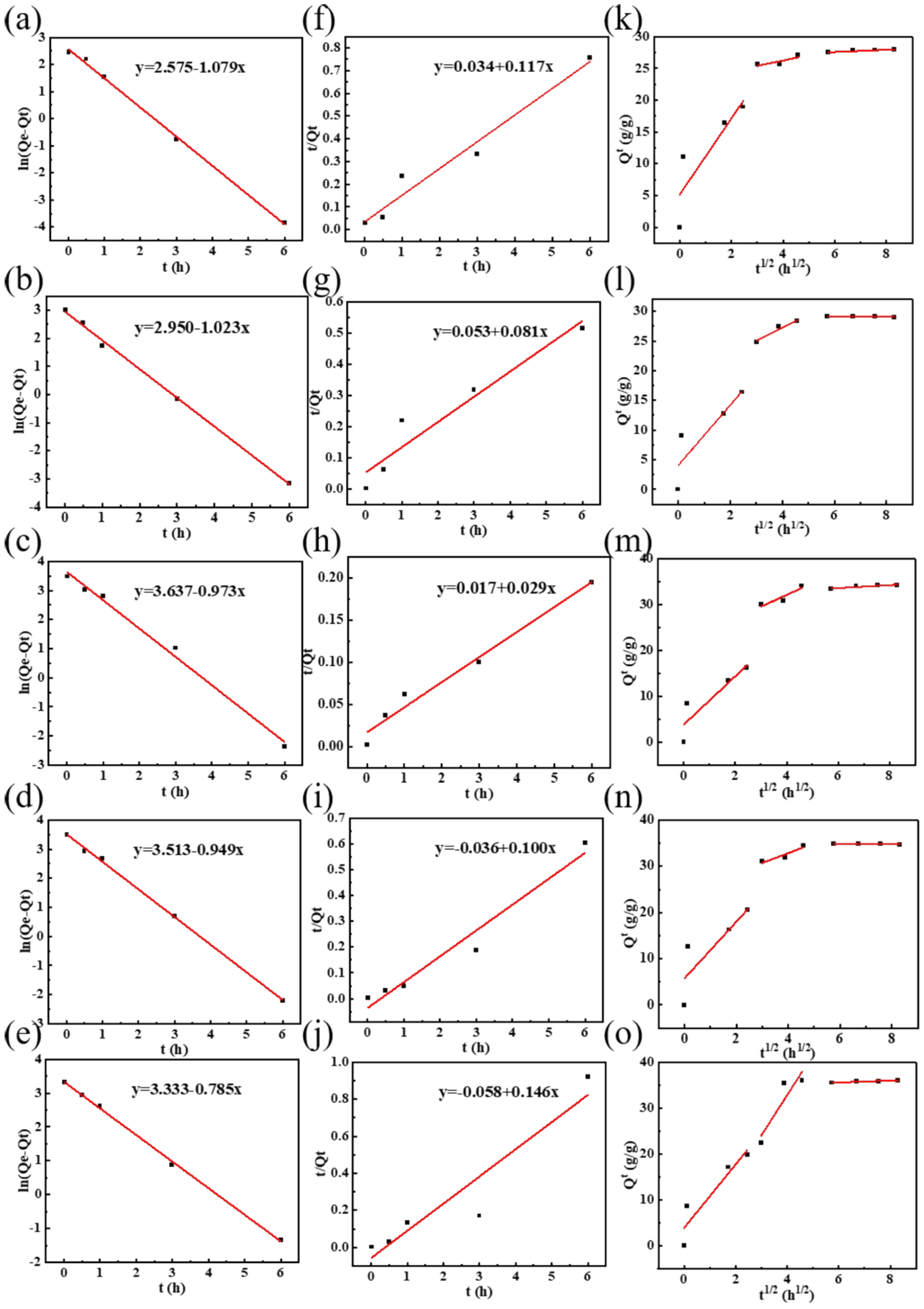

Figure S6 Fitting curves for the pseudo-first-order, pseudo-second-order, and intra-particle diffusion models of DMSP-g-PNAGA1:1

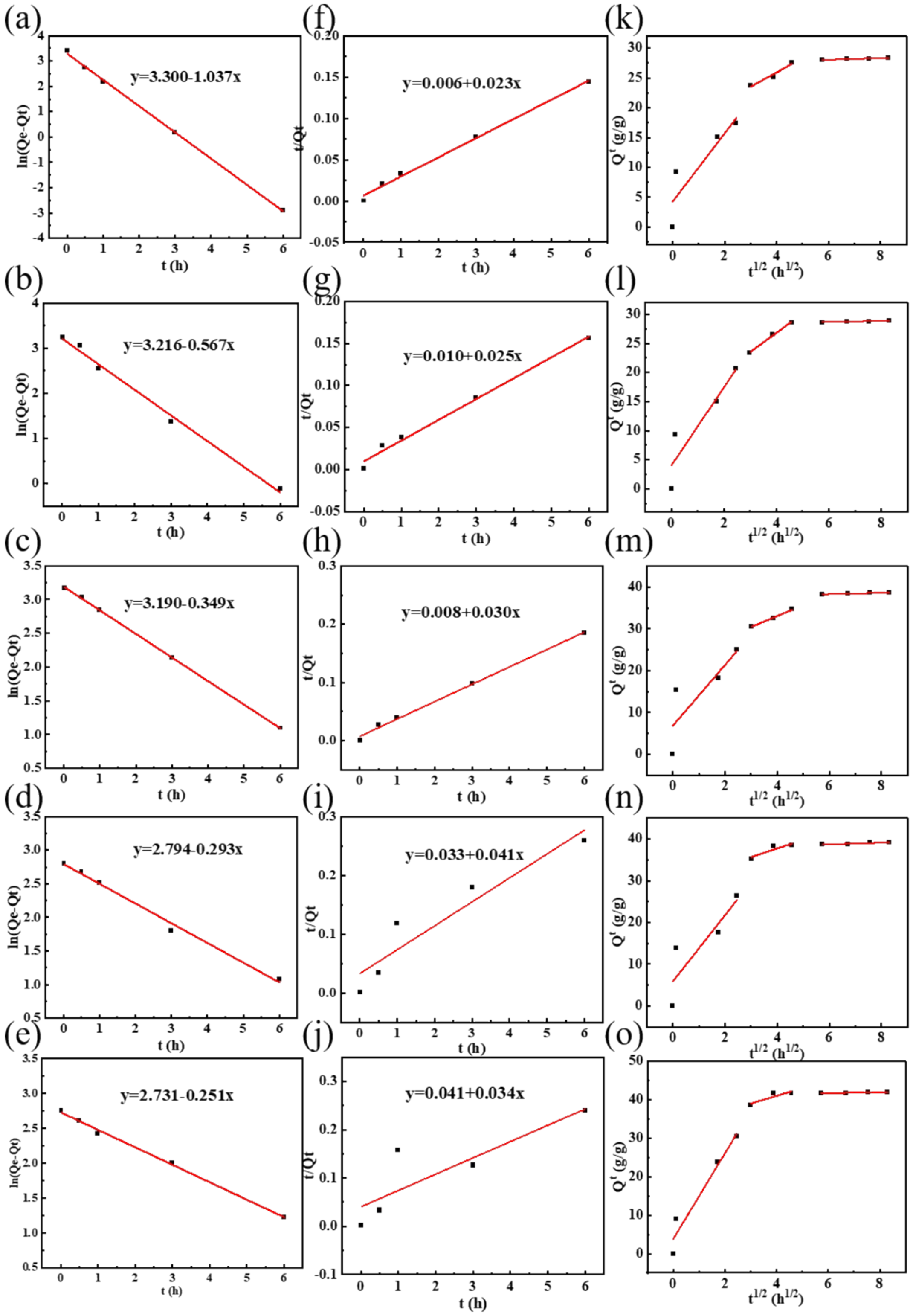

Figure S7 Fitting curves for the pseudo-first-order, pseudo-second-order, and intra-particle diffusion models of DMSP-g-PNAGA100:1

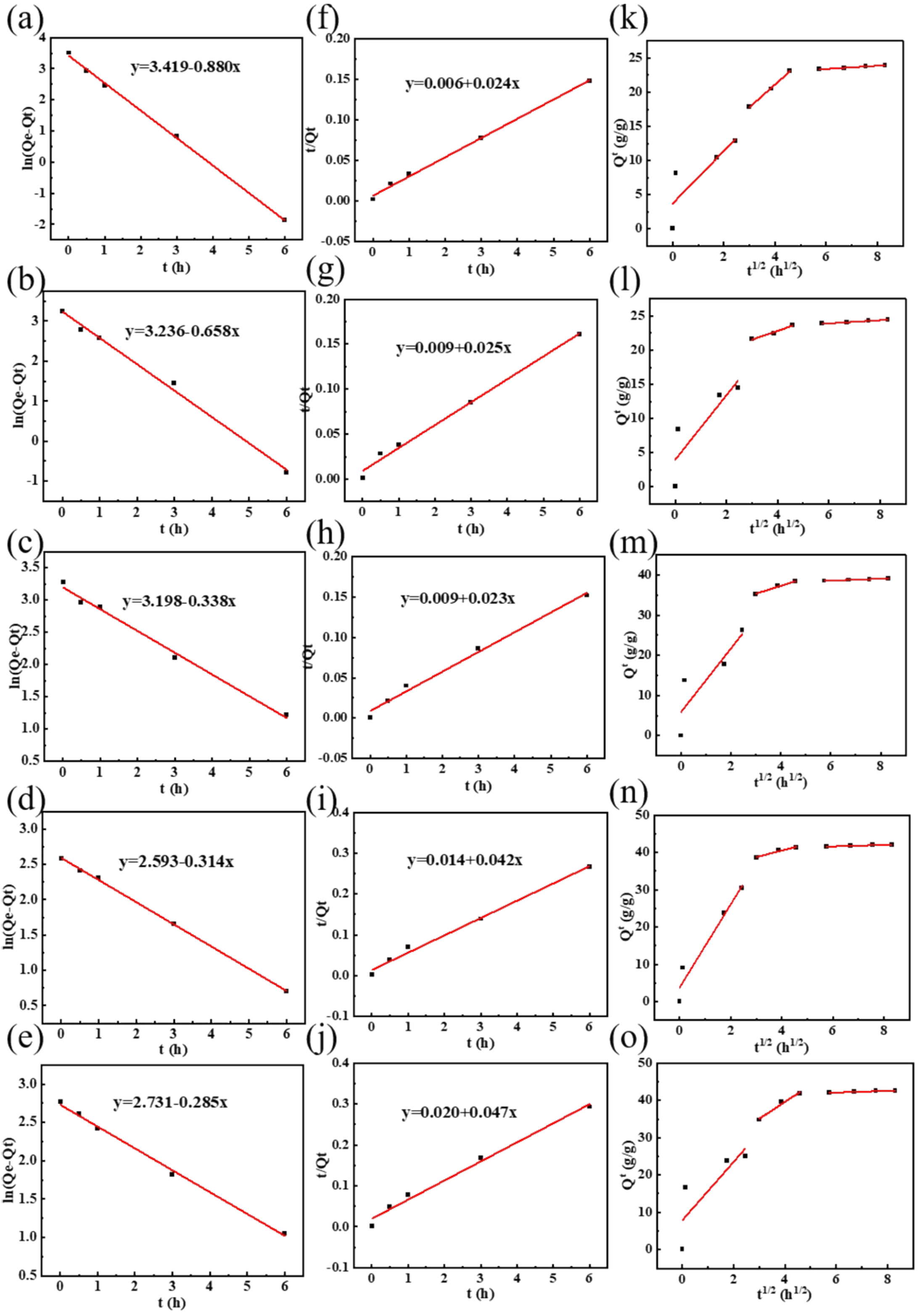

Figure S8 Fitting curves for the pseudo-first-order, pseudo-second-order, and intra-particle diffusion models of DMSP-g-PNAGA-T65

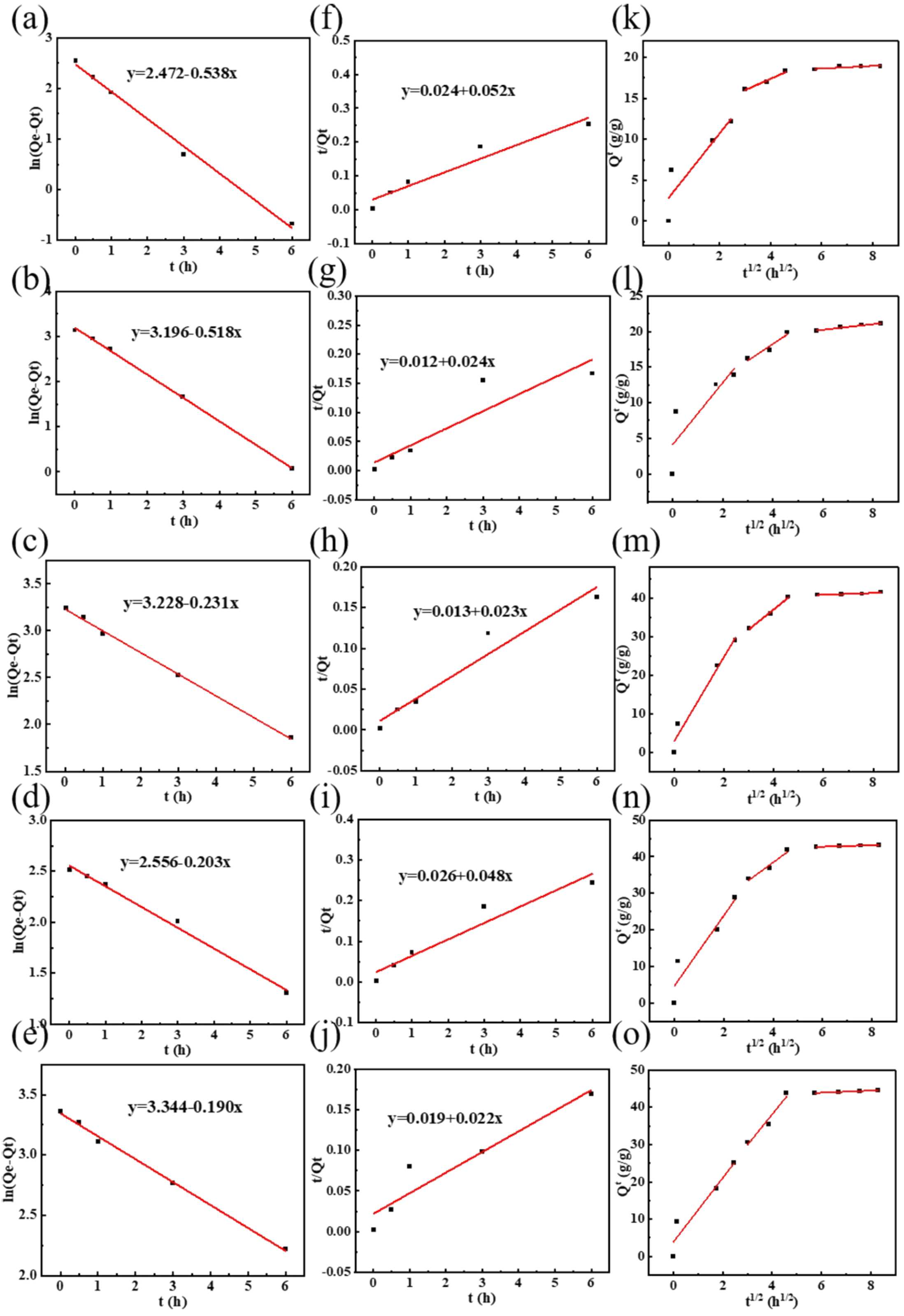

Figure S9 Fitting curves for the pseudo-first-order, pseudo-second-order, and intra-particle diffusion models of DMSP-g-PNAGA-t21

S7 ESP maps of PNAGA/ZnO adsorption complexes

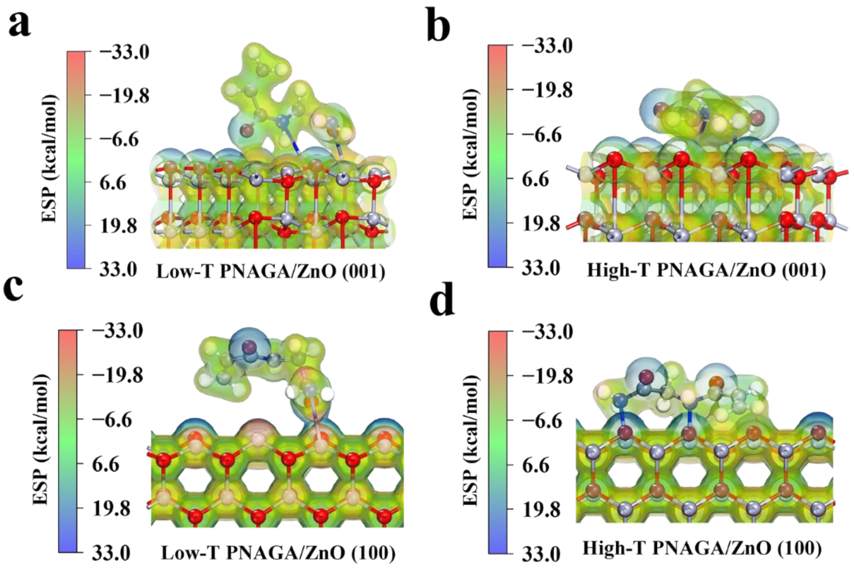

Figure S10 ESP maps of PNAGA/ZnO adsorption complexes: (a) low-T PNAGA/(100), (b) high-T PNAGA/(100), (c) low-T PNAGA/(001), and (d) high-T PNAGA/(001). The high-T PNAGA/(001) complex exhibits the most pronounced interfacial electrostatic complementarity, consistent with its strongest adsorption energy. All ESP maps were plotted using the same electrostatic potential scale.

S8 Semi-quantitative analysis of the relationship between ZnO loading and antibacterial activity

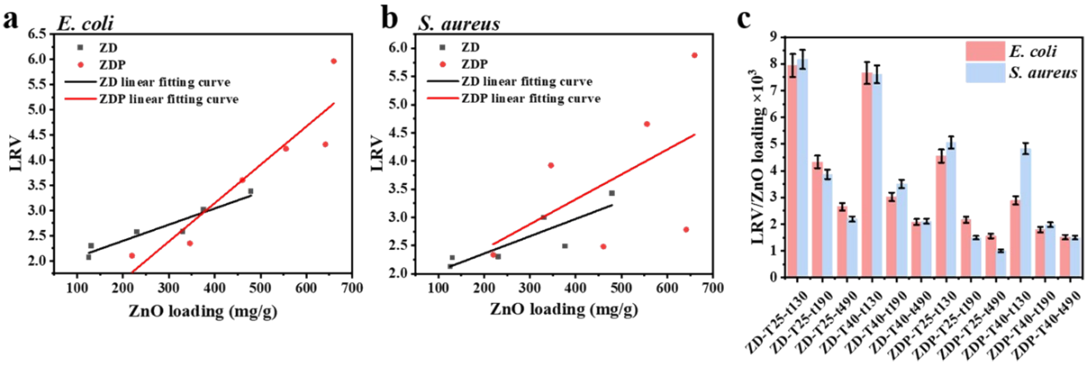

Figure S11 (a, b) Scatter plots of LRV as a function of ZnO loading. (c) Apparent loading-normalized antibacterial index (LRV/ZnO loading) for E. coli and S. aureus, respectively. The fitting lines are presented solely to indicate trends and do not imply a strict linear correlation between ZnO loading and antibacterial performance.

S9 Correlation between Zn^{2+} release and antibacterial activity

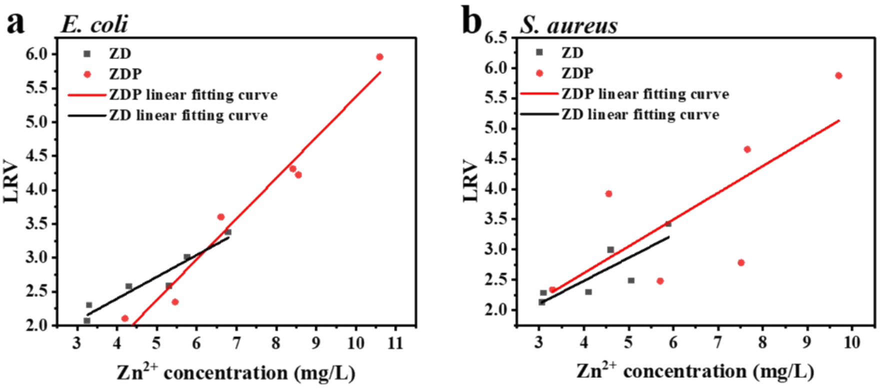

Figure. S12 Correlation between Zn^{2+} release and antibacterial activity. Scatter plots of LRV as a function of Zn^{2+} concentration at 30 min for (a) *E. coli* and (b) *S. aureus*. Each point represents one ZnO-loaded sample. Linear fitting was performed separately for the ZD and ZDP series. For *E. coli*, strong positive correlations were observed for both ZD and ZDP, with R^2 values of 0.9123 and 0.9653, respectively. For *S. aureus*, weaker correlations were observed, with R^2 values of 0.5380 and 0.7151 for ZD and ZDP, respectively. The fitting was used only for semi-quantitative trend analysis because LRV is logarithmic and Zn^{2+} release is not the sole antibacterial factor.

S10 Zeta potential of DMSP-g-PNAGA following in-situ ZnO NPs growth

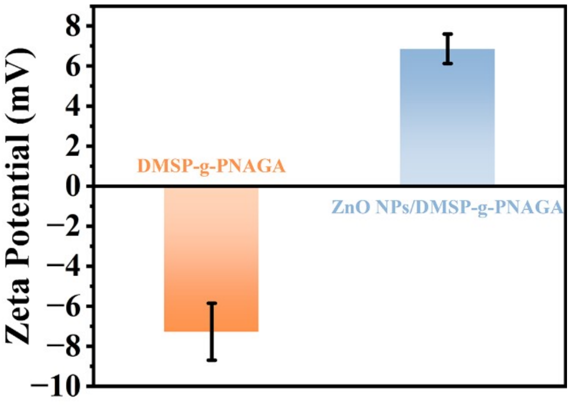

Figure S13 Zeta potentials of DMSP-g-PNAGA and ZnO NPs/DMSP-g-PNAGA. The zeta potential exhibited a substantial shift following the growth of ZnO NPs.

S11 Results of BET analysis for the isotherms used in the study

Table S1 Specific surface area and average pore size MSP, DMSP, DMSP-g-PNAGA and ZnO NPs/DMSP-g-PNAGA.

Sample	Pore size (nm)	Pore volume (cm^3/g)	Surface area (m^2/g)
MSP	12.6 ± 0.04	0.030	3.73 ± 1.51
DMSP	13.3 ± 0.27	0.021	4.11 ± 1.51
DMSP-g-PNAGA	11.6 ± 0.06	0.012	5.26 ± 0.47
ZnO NPs/DMSP-g-PNAGA	14.1 ± 0.31	0.014	5.48 ± 0.33

S12 Pseudo-first-order, pseudo-second-order, and intraparticle diffusion models fitting parameters

Table S2 Kinetic parameters for Water adsorption by DMSP-g-PNAGA1:1: linear pseudo-first-order and pseudo-second-order model

Model parameter		Linear fitting				
		**20 ℃**	**25 ℃**	**30 ℃**	**35 ℃**	**40 ℃**
**PFO**	**q_e (g/g)**	28.1215	29.9491	35.0508	35.6125	37.1471
	**q_t (g/g)**	27.9259	29. 0734	34.3336	34.9146	35.0617
	**k_1 (min^{-1})**	1.0786	1.0227	0.9735	0.9494	0.7855
	**R^2**	0.9980	0.9980	0.9927	0.9989	0.9990
	**RSS**	0.0002	0.0004	0.0007	0.0002	0.0017
**PSO**	**q_e (g/g)**	29.0651	28.5593	25.1587	30.8897	24.0697
	**q_t (g/g)**	27.9259	29.0734	34.3336	34.9146	35.0617
	**k_2 (min^{-1})**	0.1176	0.0808	0.0297	0.1002	0.1467
	**R^2**	0.9664	0.9290	0.9754	0.9635	0.8962
	**RSS**	0.0116	0.0120	0.0312	0.0451	0.1113

Table S3 Kinetic parameters for Water adsorption by DMSP-g-PNAGA1:1: intraparticle diffusion models

Parameter	Linear fitting				
	**20 ℃**	**25 ℃**	**30 ℃**	**35 ℃**	**40 ℃**
**q_e (g/g)**	24.7612	24.7652	38.4325	42.0121	43.8563
**q_t (g/g)**	27.9259	29. 0734	34.3336	34.9146	35.0617
**k_{i1}**	5.9947	5.0776	5.2493	6.1317	6.9258
**k_{i2}**	0.8453	2.2064	2.5384	2.1203	8.7645
**k_{i3}**	0.1717	0.0376	0.3255	0.0223	0.2110
**R_{i1}^2**	0.7407	0.7605	0.7963	0.7006	0.8683
**R_{i2}^2**	0.6877	0.9519	0.8537	0.8918	0.8327
**R_{i3}^2**	0.8747	0.4702	0.7342	0.9681	0.9401

Table S4 Kinetic parameters for Water adsorption by DMSP-g-PNAGA100:1: linear pseudo-first-order and pseudo-second-order model

Model parameter		Linear fitting				
		20 ℃	25 ℃	30 ℃	35 ℃	40 ℃
PFO	q_e (g/g)	28.7612	28.7652	38.4325	39.0121	41.8563
	q_t (g/g)	28.4085	28.9051	38.6731	39.0982	41.8982
	k_1 (min^{-1})	1.0372	0.5673	0.3492	0.2931	0.2506
	R^2	0.9990	0.9928	0.9998	0.9925	0.9969
	RSS	0.0021	0.0101	0.0001	0.0114	0.0146
PSO	q_e (g/g)	27.7561	29.6151	38.3121	39.6451	30.1231
	q_t (g/g)	28.4085	28.9051	38.6731	39.0982	41.8982
	k_2 (min^{-1})	0.0233	0.0248	0.0298	0.0407	0.0337
	R^2	0.9948	0.9911	0.9962	0.9014	0.7466
	RSS	0.0109	0.0134	0.0098	0.0437	0.0938

Table S5 Kinetic parameters for Water adsorption by DMSP-g-PNAGA100:1: intraparticle diffusion models

Parameter	Linear fitting				
	20 ℃	25 ℃	30 ℃	35 ℃	40 ℃
q_e (g/g)	28.7612	28.7652	38.4325	39.0121	41.8563
q_t (g/g)	28.4085	28.9051	38.6731	39.0982	41.8982
k_{i1}	5.7246	6.6913	7.2782	7.9234	11.1364
k_{i2}	2.4154	3.2817	2.6042	2.0528	1.9981
k_{i3}	0.1409	0.0914	0.1511	0.2031	0.0625
R_{i1}^2	0.7906	0.8414	0.6871	0.7604	0.9479
R_{i2}^2	0.9439	0.9964	0.9827	0.8288	0.8204
R_{i3}^2	0.9801	0.9698	0.9302	0.9468	0.9776

Table S6 Kinetic parameters for Water adsorption by DMSP-g-PNAGA-T65: linear pseudo-first-order and pseudo-second-order model

Model parameter		Linear fitting				
		**20 °C**	**25 °C**	**30 °C**	**35 °C**	**40 °C**
**PFO**	**q_e (g/g)**	24.7612	24.7652	38.4325	42.0121	43.8563
	**q_t (g/g)**	23.9464	24.4610	39.1627	42.0631	42.6428
	**k_1 (min^{-1})**	0.8803	0.6583	0.3380	0.3141	0.2849
	**R^2**	0.9989	0.9946	0.9926	0.9993	0.9958
	**RSS**	0.0015	0.0164	0.0066	0.0016	0.0082
**PSO**	**q_e (g/g)**	27.7561	29.6151	38.3121	39.6451	30.1231
	**q_t (g/g)**	23.9464	24.4610	39.1627	42.0631	42.6428
	**k_2 (min^{-1})**	0.0237	0.0254	0.0243	0.0424	0.0466
	**R^2**	0.9970	0.9922	0.9900	0.9923	0.9885
	**RSS**	0.0034	0.0107	0.0141	0.0183	0.0439

Table S7 Kinetic parameters for Water adsorption by DMSP-g-PNAGA-T65: intraparticle diffusion models

Parameter	Linear fitting				
	**20 °C**	**25 °C**	**30 °C**	**35 °C**	**40 °C**
**q_e (g/g)**	24.5681	24.6452	38.9984	42.1561	43.2481
**q_t (g/g)**	23.9464	24.4610	39.1627	42.0631	42.6428
**k_{i1}**	3.8300	4.7167	7.9235	11.1364	7.8191
**k_{i2}**	3.3237	1.2822	1.9749	1.7534	4.4941
**k_{i3}**	0.2330	0.2338	0.2341	0.2001	0.2338
**R_{i1}^2**	0.6889	0.8651	0.8720	0.9479	0.6724
**R_{i2}^2**	0.9959	0.9893	0.9867	0.9636	0.9765
**R_{i3}^2**	0.9971	0.9985	0.9971	0.9903	0.9971

Table S8 Kinetic parameters for Water adsorption by DMSP-g-PNAGA-t21: linear pseudo-first-order and pseudo-second-order model

Model parameter		Linear fitting				
		**20 °C**	**25 °C**	**30 °C**	**35 °C**	**40 °C**
**PFO**	**q_e (g/g)**	18.9311	21.1142	41.5184	43.1857	44.6024
	**q_t (g/g)**	18.9482	21.0661	41.5338	43.1578	44.5714
	**k_1 (min^{-1})**	0.5383	0.5184	0.2306	0.2033	0.1901
	**R^2**	0.9992	0.9977	0.9985	0.9982	0.9950
	**RSS**	0.0008	0.0022	0.0003	0.0003	0.0009
**PSO**	**q_e (g/g)**	20.4241	23.1422	36.4251	41.6242	40.1424
	**q_t (g/g)**	18.9482	21.0661	41.5338	43.1578	44.5714
	**k_2 (min^{-1})**	0.0521	0.0244	0.0239	0.0479	0.0218
	**R^2**	0.9384	0.9365	0.8911	0.9574	0.8822
	**RSS**	0.8992	0.6056	1.8330	0.9560	2.6112

Table S9 Kinetic parameters for Water adsorption by DMSP-g-PNAGA-t21: intraparticle diffusion models

Parameter	Linear fitting				
	**20 °C**	**25 °C**	**30 °C**	**35 °C**	**40 °C**
**q_e (g/g)**	24.7612	24.7652	38.4325	42.0121	43.8563
**q_t (g/g)**	18.9482	23.0661	41.5338	43.1578	44.5714
**k_{i1}**	3.9394	4.3563	10.9057	9.5850	8.5803
**k_{i2}**	1.3882	2.3227	5.1892	4.9029	8.1826
**k_{i3}**	0.1644	0.3998	0.2449	0.1954	0.2809
**R_{i1}^2**	0.8028	0.7039	0.9654	0.8811	0.9039
**R_{i2}^2**	0.9796	0.9406	0.9913	0.9521	0.9559
**R_{i3}^2**	0.7677	0.9426	0.8267	0.9499	0.9974

S13 Linear fitting was performed using ZnO loading as the independent variable and LRV as the dependent variable

Table S10 Linear fitting parameters for the relationship between ZnO loading and LRV.

Bacterial strain	Series	Fitting equation	Slope	Intercept	R^2
E. coli	ZD	y=ax+b	0.0032	1.7555	0.9159
E. coli	ZDP	y=ax+b	0.0076	0.1004	0.8593
S. aureus	ZD	y=ax+b	0.0031	1.7447	0.7522
S. aureus	ZDP	y=ax+b	0.0044	1.5541	0.2973

S14 Linear fitting parameters for the relationship between Zn^{2+} release and LRV

Table S11 Linear fitting parameters for the relationship between Zn^{2+} release and LRV

Bacterial strain	Series	Fitting equation	Slope	Intercept	R^2
E. coli	ZD	y=ax+b	0.3215	1.1126	0.9123
E. coli	ZDP	y=ax+b	0.5999	-0.6228	0.9653
S. aureus	ZD	y=ax+b	0.3803	0.9645	0.7151
S. aureus	ZDP	y=ax+b	0.4418	0.8476	0.5380

S15 Variation in r values for multiple DMSP and DMSP-g-PNAGA samples

Table S12 r values of DMSP and DMSP-g-PNAGA samples at reaction temperatures of 25 °C and 40 °C and times of 130, 190, and 490 min

Sample	r
ZD-T25-t130	1.2
ZD-T25-t190	1.1
ZD-T25-t490	1.3
ZD-T40-t130	1.1
ZD-T40-t190	1.2
ZD-T40-t490	1.2
ZDP-T25-t130	0.8
ZDP-T25-t190	0.9
ZDP-T25-t490	0.9
ZDP-T40-t130	1.1
ZDP-T40-t190	1.3
ZDP-T40-t490	1.4

Supplementary References

1 T. Tian, S. Tu, A. Xu, S. Yin, A. L. Oechsle, T. Xiao, A. Vagias, J. Eichhorn, J. Suo, Z. Yang, S. Bernstorff and P. Müller-Buschbaum, *Adv. Funct. Mater.*, DOI:10.1002/adfm.202311793.

2 X. Wu, Y. Liu, M. Li, B. Li, X. Mao, Q. Wang, X. Tang, H. Zhang, L. Peng and X. Gao, *Chem. Eng. J*, DOI:10.1016/j.cej.2024.153757.

3 G. E. Dawwam, M. T. Al-Shemy and A. S. El-Demerdash, *Sci. Rep.*, 2022, **12**, 1–18.

4 G. Sun, Y. Chen, X. Xia, Y. Wang, J. Zeng, J. Zhu, L. Gao, L. Peng, L. Wang, X. Kong and Y. Jin, DOI:10.1021/jacs.5c15840.

5 S. Wang, Z. Zhao, L. Jia, X. Guo, R. Yang, Q. Deng and R. Sun, DOI:10.1021/acssensors.4c01374.

6 Y. Zhang, C. Wang, F. Gong, P. Wang, U. Guharoy, C. Yang, H. Zhang, S. Fang and J. Liu, *J. Hazard. Mater.*, 2020, **388**, 122069.
